# Phytotoxic Azaphilones From the Mangrove-Derived Fungus *Penicillium sclerotiorum* HY5

**DOI:** 10.3389/fmicb.2022.880874

**Published:** 2022-04-19

**Authors:** Wei Wang, Mei Wang, Xian-Bo Wang, Yi-Qiang Li, Ji-Lin Ding, Ming-Xian Lan, Xi Gao, Dong-Lin Zhao, Cheng-Sheng Zhang, Guo-Xing Wu

**Affiliations:** ^1^College of Plant Protection, Yunnan Agricultural University, Kunming, China; ^2^Tobacco Research Institute of Chinese Academy of Agricultural Sciences, Qingdao, China; ^3^Guizhou Tobacco Company, Zunyi Branch, Zunyi, China

**Keywords:** azaphilones, phytotoxicity, bioherbicide, weeds, *Penicillium sclerotiorum*

## Abstract

Mangrove is a unique marine ecosystem growing in the intertidal zone of tropical and subtropical coast, with the characteristics of hypoxia tolerance, high salinity, and high humidity. In order to discover novel leading compounds with potent phytotoxicity, seven pairs of azaphilones *E/Z* isomers, isochromophilone H (**1a**/**1b**), sclerotiorins A and B (**2a**/**2b** and **3a/3b**), ochlephilone (**4a**/**4b**), isochromophilone IV (**5a**/**5b**), isochromophilone J (**6a**/**6b**), and isochromophilone I (**7a**/**7b**), were isolated from the culture broth of the mangrove-derived fungus, the *Penicillium sclerotiorum* HY5, by various chromatographic methods. Among them, **1a**, **1b**, **2a**, **3a**, **4a**, **5a, 6a**, and **6b** were new compounds. Their chemical structures and absolute configurations were elucidated based on high resolution electrospray ionization mass spectroscopy (HRESIMS), 1D/2D nuclear magnetic resonance (NMR) spectroscopic analysis, and comparisons of electronic circular dichroism (ECD) data. Compounds **3**, **4**, and **7** exhibited potent phytotoxicity against the growth of radicle and plumule on *Amaranthus retroflexus* L., with EC_50_ values ranging from 234.87 to 320.84 μM, compared to the positive control glufosinate-ammonium, with EC_50_ values of 555.11 μM for radicle, and 656.04 μM for plumule. Compounds **4** and **7** also showed inhibitory effects on the growth of velvetleaf (*Abutilon theophrasti* Medikus), with EC_50_ values ranging from 768.97 to 1,201.52 μM. This study provides new leading compounds for the research and development of marine-derived bioherbicides.

## Graphical Abstract

Representative phytotoxic azaphilones from *Penicillium sclerotiorum* HY5. 
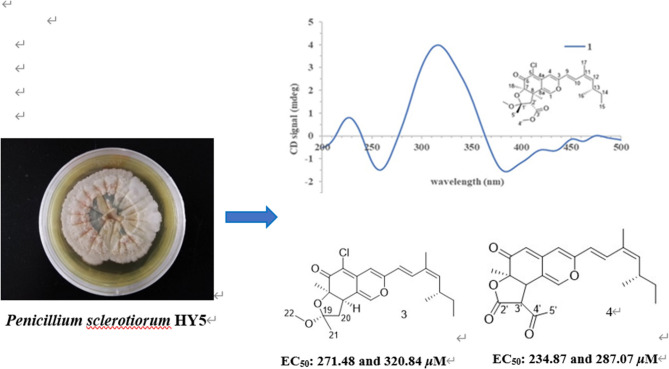

## Introduction

Weeds are common, pernicious, and troublesome plant species, which can cause serious yield reduction and inferior quality in crop production. It is estimated that the production loss caused by weeds is approximately 34% of the crop yield worldwide (Harding and Raizada, 2015; Shi et al., 2020). In the present situation, control weeds in farmland on a global scale are mainly dependent on chemical treatments (Travaini et al., 2016; Vurro et al., 2018); however, there are many long-term problems with the intensive application of agrochemical herbicides, such as environmental pollution, pesticide residue accumulation, and the emergence of weed resistance, which increase the difficulty of weeds control management (Kim et al., 2020; Shi et al., 2020). With the continuous discoveries of a great number of biocontrol microbial resources, exploration of microbes with excellent biological activity from extreme conditions such as marine-derived microorganisms, in recent years, have attracted much attention of scientists (Shen et al., 2020). Marine surroundings provide abundant microbial resources because of their geographic and climatic characteristics. These features make it become an important field for discovering bioactive natural products with agricultural applications (Yang et al., 2015; Carroll et al., 2020).

Azaphilones are a class of fungi-derived polyketide secondary metabolites with novel structures having an oxabicyclic skeleton and can be divided into 18 different categories, which have numerous chiral centers and flexible side chains (Gao et al., 2013; Makrerougras et al., 2017). Previous reports have shown that they exhibited broad-spectrum activities in many biological tests, including antimicrobial, antiviral, anti-inflammatory, antioxidant, cytotoxic, hypoglycemic, and nematocidal activities (Luo et al., 2018; Wang et al., 2020). More than 430 azaphilones, isolated from both marine and terrestrial fungi, have been reported until 2019, representing an important class of natural products (Qian et al., 2019). However, most of the azaphilones were utilized for drug development, and their agricultural bioactivities need to be explored.

**Figure 1:**
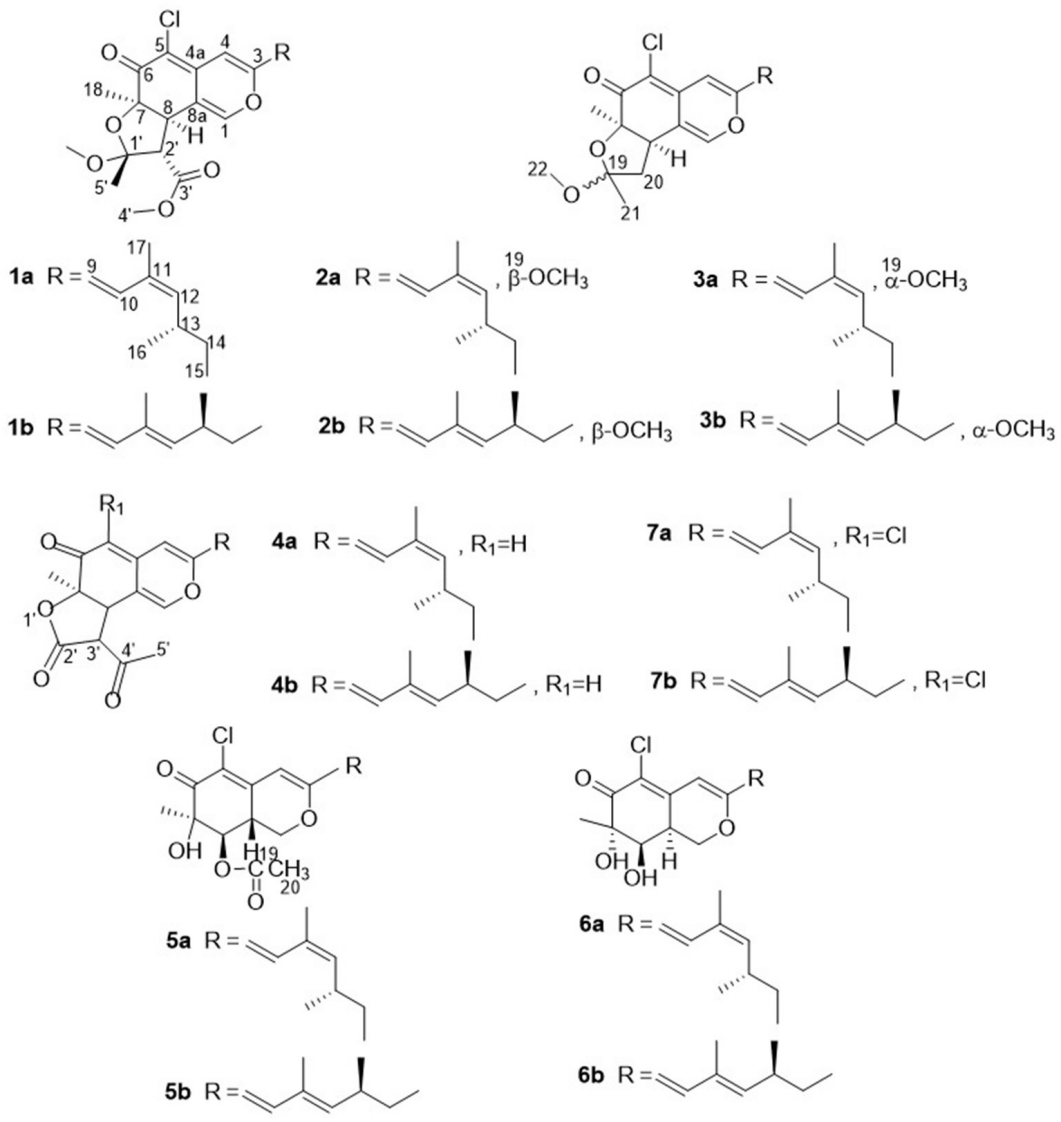
Chemical structures of compounds **1–7**.

During our ongoing search for phytotoxic compounds with agricultural applications (Huang et al., 2018; Zhao et al., 2019, 2020), the mangrove-derived strain *Penicillium sclerotiorum* HY5 attracted our attention because its culture extracts demonstrated potent phytotoxicity toward *Amaranthus retroflexus* L., and the high-performance liquid chromatography (HPLC) profile highlighted a rich array of ultraviolet absorption peaks similar to that of azaphilones. Further chemical investigation on the fungal extracts resulted in the isolation of seven pairs of azaphilones *E/Z* isomers, isochromophilone H (**1a** and **1b**), sclerotiorin A (**2a** and **2b**), sclerotiorin B (**3a** and **3b**), ochlephilone (**4a** and **4b**), isochromophilone IV (**5a** and **5b**), isochromophilone J (**6a** and **6b**), and isochromophilone I (**7a** and **7b**; Figure 1). Here, we report the isolation, structural elucidation, and phytotoxic evaluation of isolated azaphilones.

## Materials and Methods

### General Experimental Procedures

Optical rotations were measured at 25°C using a JASCO P-1020 digital polarimeter (JASCO Ltd., Tokyo, Japan). The UV spectra were determined on a Techcomp UV2310II spectrophotometer (Techcomp, Ltd., Shanghai, China). Electronic circular dichroism (ECD) spectra were acquired with a JASCO J-815 CD spectrometer (JASCO Ltd., Tokyo, Japan) at 25°C. The NMR spectra were recorded on a DD2 NMR spectrometer (Agilent Technologies, Santa Clara, CA, USA; 500 MHz for ^1^H and 125 MHz for ^13^C) and a JNM-ECP NMR spectrometer (JEOL, Japan; 600 MHz for ^1^H and 150 MHz for ^13^C) using tetramethylsilane (TMS) as an internal standard, and CDCl_3_ as solvent. The Electrospray ionization mass spectrometry (ESIMS) was taken on a Micromass Q-TOF spectrometer (Waters, Ltd., Milford, Massachusetts, USA), and high-resolution ESIMS spectra were collected on a Thermo Scientific LTQ Orbitrap XL spectrometer (Thermo Fisher Scientific, Waltham, MA, USA). Semipreparative HPLC was conducted on a Waters e2695 separation system (Milford, MA, USA), equipped with a Waters 2998 photodiode array detector and a Waters X-Bridge C_18_ (5 μm, 10 × 250 mm) preparative column, and the flow rate was 2 mL/min. Column chromatography (CC) was performed using Silica gel (100–200, 200–300 mesh; Qingdao Marine Chemical Inc., Qingdao, China), Sephadex LH-20 (GE Healthcare, Pittsburgh, PA, USA), and octadecylsilyl silica gel (ODS) (40–63 μm, merck, MA, USA). Precoated silica gel plates (Yantai Zi fu Chemical Group Co., Yan Tai, China; GF254) were used for thin layer chromatography (TLC) analysis. Spots were detected by UV light (254 nm) and colored by spraying heated silica gel plates with 12% H_2_SO_4_ in H_2_O containing saturated vanillin.

### Fungal Material

The studied strain was isolated from an unidentified mangrove sample collected from coastal marine habitats of the South China Sea, Haikou, China, in May 2017. This strain was identified as *P. sclerotiorum* HY5 by amplifying and analyzing its internal transcribed spacer (ITS) sequence, as well as morphological features. The ITS sequence of this fungus was deposited at the GenBank database (accession number MG827186), and the isolated strain was preserved in the Marine Agriculture Research Center, Tobacco Research Institute of Chinese Academy of Agricultural Sciences, Qingdao, China.

### Fermentation, Extraction, and Isolation

The purified fungus was grown on PDA plates for 5 days at 28°C. The mycelial disc were inoculated into 500 mL Erlenmeyer flasks containing 200 mL of potato dextrose water (PDW) medium with 3% salinity and shaken on a rotary shaker (175 rpm) at 28°C for 3 days to obtain seed culture. Aliquots (5 mL) of the seed culture were transferred into 1,000 mL Erlenmeyer flasks containing 400 mL of production media, consisting of PDW medium with 3% salinity for 30 days at 28°C, and the fermentation scale was 80 L in total. After fermentation, the culture broth was filtered to separate the culture media and mycelia. The culture broth was extracted three times with equal volumes of EtOAc. The mycelia were mechanically broken, and then, extracted ultrasonically twice with a mixture (1:1, v/v) of dichloromethane (CH_2_Cl_2_) and methanol (MeOH), and concentrated in vacuo to obtain an aqueous solution, which was further extracted thrice with equivalent EtOAc. The culture broth and mycelia extracts were combined and evaporated under reduced pressure to yield EtOAc extract (74.61 g), which was then subjected to vacuum liquid chromatography (VLC) on silica gel using a step gradient elution of EtOAc–petroleum ether from 0 to 100% and 10 to 50% MeOH–EtOAc to afford six fractions (Fr.1–Fr.6) based on their TLC profiles. The Fr.2 was chromatographed repeatedly on silica gel CC eluting with mixtures of EtOAc–petroleum ether (10–50%, v/v) to give three subfractions (Fr.21–Fr.23). The Fr.23 was first fractionated *via* reverse silica *gel* CC eluting with a gradient MeOH–H_2_O (from 50:50 to 100:0, v/v), and then, separated by Sephadex LH-20 CC eluting with CH_2_Cl_2_-MeOH (1:1, v/v) to obtain two subfractions (Fr.231–Fr.232). The Fr.231 was then chromatographed repeatedly with a gradient elution of EtOAc–petroleum ether (from 0 to 100%, v/v) and MeOH–EtOAc (from 10 to 50%, v/v) to give four subfractions (Fr.2311–Fr.2314). The Fr.2311 was applied to semipreparative HPLC separation using an isocratic elution of MeOH–H_2_O with 0.1% trifluoroacetic acid (TFA) (75% MeOH in H_2_O, v/v) to afford compound **7** (119.8 mg). The Fr.2313 was subjected to semipreparative HPLC using an isocratic elution of 70% MeCN in H_2_O to yield compound **1** (26.8 mg). Following the same procedures, Fr.3 was also subjected to silica gel CC using a step gradient elution of EtOAc–petroleum ether (0 to 100%, v/v) and MeOH–EtOAc (10 to 50%, v/v) to give two subfractions (Fr.31–Fr.32). The Fr.31 was separated by octadecylsilyl (ODS) CC (MeOH–H_2_O; from 50:50 to 100:0, v/v), and then, subjected to Sephadex LH-20 CC eluting with CH_2_Cl_2_-MeOH (1:1, v/v) to obtain two subfractions (Fr.311–Fr.312). The Fr.311 was further purified by using semipreparative HPLC with isocratic MeOH–H_2_O (75:25, v/v) as mobile phase to yield compound **5** (166.3 mg). The Fr.312 was subjected to semipreparative HPLC using MeOH in H_2_O (25%) to obtain compounds **2** (28.4 mg) and **3** (28.1 mg). The Fr.32 was eluted using a MeOH–H_2_O gradient system (from 50:50 to 100:0, v/v) and sequentially subjected to Sephadex LH-20 CC (CH_2_Cl_2_-MeOH, 1:1, v/v) to give three subfractions (Fr.321–Fr.323). The Fr.322 was applied to semi-preparative HPLC (60% MeCN in H_2_O) to afford compound **4** (164 mg). The Fr.323 was purified by semipreparative HPLC (75% MeOH in H_2_O) to generate compound **6** (63 mg). In order to achieve the requirements of the NMR test, the purities of all the isolated compounds were >95% based on the peak area normalization methods.

*Isochromophilone H (**1**)**:*** yellow amorphous powder; [α]${}_{\text{D}}^{25.0}$ −15.9 (*c*, 0.46, MeOH); UV(MeOH) λ_max_ (log ε) 202 (3.38), 248 (3.23), and 387 (3.35) nm; ECD (*c* 1.08 mM, MeOH) λ_max_ (Δε) 223 (+0.34), 257 (−0.51), 312 (+1.18), 385 (−0.51) nm; ^1^H, and ^13^C NMR data (Tables 1, 2); HRESIMS *m/z* 463.1892 [M+H] ^+^(calculated for C_25_H_31_O_6_Cl, 463.1882).

**Table 1:** ^1^H nuclear magnetic resonance (NMR) data of compounds **1–6** (chloroform-*d*, δ in ppm, *J* in Hz).

**No**.	**1a**	**1b**	**2a**	**3a**	**4a**	**5a**	**6a**	**6b**
1α	7.56, s	7.53, s	7.12, s	7.21, s	7.43, s	3.87, dd (10.8,13.2)	4.30, dd (11.4,12.6)	4.27, dd (11.5, 13.0)
1β						4.43, dd (4.8,10.8)	4.55, dd (4.8, 11.4)	4.52, dd (5.0, 11.5)
4	6.53, s	6.51, s	6.52, s	6.53, s	6.11, s	6.14, s	6.12, s	6.10, s
5					5.42, d (1.0)			
8	3.80, d (12.6)	3.79, d (12.6)	3.06, dd (10.0,10.0)	3.43, dd (7.5,13.0)	3.85, d (12.0)	5.03, d (10.2)	4.14, d (3.0)	4.12, d (3.0)
8a						3.49, ddd (4.8,10.2,13.2)	3.09 ddd (3.0, 4.8, 12.6)	3.07 ddd (3.0, 5.0, 13.0)
9	6.14, d (15.6)	6.05, d (15.6)	6.15, d (16.0)	6.15, d (15.5)	6.04, d (15.5)	6.09, d (15.0)	6.09, d (15.6)	6.00, d (15.5)
10	7.41, d (15.6)	7.02, d (15.6)	7.36, d (15.5)	7.39, d (15.5)	7.36, d (15.5)	7.36, d (15.0)	7.38, d (15.6)	7.01, d (16.0)
12	5.49, d (10.2)	5.63, d (10.2)	5.47, d (10.0)	5.48, d (10.0)	5.48, d (10.0)	5.46, d (10.2)	5.47, d (9.6)	5.63, d (10.0)
13	2.64, m	2.48, m	2.64, m	2.65, m	2.59, m	2.60, m	2.66, m	2.49, m
14	1.33,1.44, m	1.33,1.44, m	1.31, 1.43, m	1.33, 1.40, m	1.31, 1.44, m	1.30, 1.40, m	1.31, 1.43, m	1.31, 1.43, m
15	0.85, t (7.2)	0.85, t (7.2)	0.85, t (7.5)	0.84, t (7.5)	0.84, t (7.5)	0.85, t (7.2)	0.86, t (7.8)	0.84, t (7.0)
16	1.01, d (6.6)	1.00, d (6.6)	1.00, d (7.0)	1.00, d (6.5)	1.00, d (7.0)	0.99, d (7.2)	0.99, d (6.6)	0.99, d (7.0)
17	1.89, s	1.82, s	1.89, d (1.0)	1.89, d (1.0)	1.87, d (1.0)	1.88, s	1.88, s	1.80, s
18	1.45, s	1.45, s	1.37, s	1.42, s	1.59, s	1.43, s	1.38, s	1.37, s
20			2.14, dd (10.0,13.0)	2.07, dd (7.5,12.5)		2.22, s		
			2.39, dd (10.0,13.0)	2.15, dd (7.5,12.5)				
21			1.46, s	1.44, s				
22			3.21, s	3.33, s				
2'	3.04, d (12.6)	3.04, d (12.6)						
3'					3.79, d (12.0)			
4'	3.74, s	3.73, s						
5'	1.58, s	1.57, s			2.47, s			
1'-OCH_3_	3.32, s	3.31, s						
7-OH						2.16, s	4.08, s	
8-OH							2.78, s	

*Recorded at 600 MHz (**1a**, **1b**, **5a**, **6a**, and **6b**). Recorded at 500 MHz (**2a**, **3a**, and **4a**)*.

**Table 2:** ^13^C NMR data of compounds **1–6** (chloroform-*d*, δ in ppm).

**Position**	**1a**	**1b**	**2a**	**3a**	**4a**	**5a**	**6a**	**6b**
1	146.0, CH	146.0, CH	142.8, CH	143.6, CH	147.4, CH	67.8, CH_2_	68.2, CH_2_	68.2, CH_2_
3	157.7, C	157.9, C	157.0, C	157.5, C	156.8, C	163.1, C	162.7, C	162.9, C
4	105.8, CH	105.1, CH	106.0, CH	105.6, CH	108.2, CH	102.2, CH	102.8, CH	102.1, CH
4a	114.2, C	114.2, C	138.8, C	139.4, C	144.5, C	145.5, C	145.4, C	145.6, C
5	109.3, C	109.3, C	110.9, C	110.0, C	106.3, CH	118.9, C	115.6, C	115.4, C
6	188.4, C	188.4, C	188.6, C	189.4, C	191.1, C	187.0, C	192.8, C	192.7, C
7	83.6, C	83.6, C	83.9, C	84.8, C	82.8, C	74.9, C	77.3, C	77.3, C
8	44.6, CH	44.6, CH	44.6, CH	43.5, CH	42.8, CH	73.1, CH	73.6, CH	73.6, CH
8a	140.3, C	140.3, C	117.1, C	116.3, C	113.9, C	35.6, CH	36.9, CH	36.9, CH
9	118.8, CH	116.4, CH	119.2, CH	119.0, CH	118.4, CH	121.2, CH	121.3, CH	118.9, CH
10	133.4, CH	141.8, CH	132.6, CH	133.0, CH	133.1, CH	133.5, CH	133.4, CH	141.9, CH
11	129.8, C	131.9, C	129.9, C	129.9, C	129.7, C	130.2, C	130.2, C	132.2, C
12	145.1, CH	147.6, CH	144.5, CH	144.8, CH	145.0, CH	144.8, CH	144.7, CH	147.1, CH
13	34.0, CH	35.0, CH	34.0, CH	34.0, CH	34.0, CH	34.0, CH	33.9, CH	34.9, CH
14	30.2, CH_2_	30.1, CH_2_	30.3, CH_2_	30.2, CH_2_	30.2, CH_2_	30.3, CH_2_	30.3, CH_2_	30.1, CH_2_
15	12.0, CH_3_	11.9, CH_3_	12.0, CH_3_	12.0, CH_3_	12.0, CH_3_	12.0, CH_3_	12.0, CH_3_	11.9, CH_3_
16	20.9, CH_3_	20.2, CH_3_	20.9, CH_3_	20.9, CH_3_	20.9, CH_3_	20.9, CH_3_	21.0, CH_3_	20.3, CH_3_
17	20.1, CH_3_	12.4, CH_3_	20.1, CH_3_	20.1, CH_3_	20.1, CH_3_	20.1, CH_3_	20.2, CH_3_	12.4, CH_3_
18	24.6, CH_3_	24.6, CH_3_	24.1, CH_3_	24.7, CH_3_	23.2, CH_3_	20.7, CH_3_	23.4, CH_3_	23.4, CH_3_
19			106.4, C	105.6, C		170.3, C		
20			47.1, CH_2_	45.5, CH_2_		20.6, CH_3_		
21			22.6, CH_3_	21.7, CH_3_				
22			49.0, CH_3_	48.9, CH_3_				
1′	105.6, C	105.5, C						
2'	58.2, CH	58.3, CH			168.5, C			
3'	169.3, C	169.2, C			57.3, CH			
4'	52.2, CH_3_	52.2, CH_3_			200.0, C			
5'	21.5, CH_3_	21.5, CH_3_			30.2, CH_3_			
1'-OCH_3_	49.2, CH_3_	49.2, CH_3_						

*Recorded at 150 MHz (**1a**, **1b**, **5a**, **6a**, and **6b**). Recorded at 125 MHz (**2a**, **3a**, and **4a**)*.

*Sclerotiorin A (**2**)**:*** yellow amorphous powder; [α]${}_{\text{D}}^{25.0}$ +3.1 (*c*, 0.37, MeOH); UV(MeOH) λ_max_ (log ε) 200 (3.46), 249 (3.34), and 390 (3.47) nm; ECD (*c* 1.23 mM, MeOH) λ_max_ (Δε) 238 (+0.84), 258 (−0.94), 312 (+3.82), and 387 (−1.13) nm; ^1^H and ^13^C NMR data (Tables 1, 2); HRESIMS *m/z* 405.1832 [M+H] ^+^(calculated for C_23_H_29_O_4_Cl, 405.1827).

*Sclerotiorin B (**3**)**:*** yellow amorphous powder; [α]${}_{\text{D}}^{25.0}$ +28.8 (*c*, 0.34, MeOH); UV(MeOH) λ_max_ (log ε) 201 (3.72), 250 (3.67), and 392 (3.82) nm; ECD (*c*, 0.62 mM, MeOH) λ_max_ (Δε) 235 (+1.84), 259 (−1.97), 313 (+8.85), and 381 (−2.37) nm; ^1^H and ^13^C NMR data (Tables 1, 2); HRESIMS *m/z* 405.1835 [M+H] ^+^(calculated for C_23_H_29_O_4_Cl, 405.1827).

*Ochlephilone (**4**)**:*** orange amorphous powder; [α]${}_{\text{D}}^{25.0}$ +195.3 (*c*, 0.54, MeOH); UV (MeOH) λ_max_ (log ε) 204 (3.89), 250 (4.04), 395 (4.25) nm; ECD (*c* 1.30 mM, MeOH) λ_max_ (Δε) 239 (+2.84), 267 (−0.56), 310 (+6.11), and 340 (+7.14) nm; ^1^H and ^13^C NMR data (Tables 1, 2); HRESIMS *m/z* 383.1856 [M+H] ^+^(calculated for C_23_H_26_O_5_, 383.1853).

*Isochromophilone IV (**5**)**:*** yellow amorphous powder; [α]${}_{\text{D}}^{25.0}$ −69.1 (*c* 0.44, MeOH); UV(MeOH) λ_max_ (log ε) 200 (3.33), 265 (2.98), 388 (3.83) nm; ECD (*c* 0.63 mM, MeOH) λ_max_ (Δε) 257 (−5.03), 285 (+1.04), 325 (+0.32), 383 (−7.82) nm; ^1^H and ^13^C NMR data (Tables 1, 2); HRESIMS *m/z* 395.1627 [M+H] ^+^(calculated for C_21_H_27_O_5_Cl, 395.1620).

*Isochromophilone J (**6**)**:*** yellow amorphous powder; [α]${}_{\text{D}}^{25.0}$ +113.3 (*c*, 0.15, MeOH); UV(MeOH) λ_max_ (log ε) 208 (3.38), 265 (3.15), and 390 (3.94) nm; ECD (*c* 1.42 mM, MeOH) λ_max_ (Δε) 214 (−1.87), 255 (+3.47), 321(−0.34), and 388 (+1.88) nm; ^1^H and ^13^C NMR data (Tables 1, 2); HRESIMS *m/z* 353.1524 [M+H] ^+^(calculated for C_19_H_25_O_4_Cl, 353.1514).

### Phytotoxicity Bioassays

Phytotoxicity was evaluated by seed germination methods. The bioassay experiments were performed on representative weeds in farmlands, including two types of grass [wild oat (*Avena fatua* L.), ryegrass (*Lolium perenne* L.)] and two broadleaf species [redroot amaranth (*A. retroflexus* L.), and velvetleaf (*Abutilon theophrasti* Medikus)], based on previously reported assay methods with some modifications (Travaini et al., 2016; Adetunji et al., 2018).

The tested seeds were pre-incubated in 9-cm diameter Petri dishes with 5 mL of distilled water for about 5 h at 25°C. After that period, the seeds of the target weeds were disinfected with 5% sodium hypochlorite for 10 min and rinsed with distilled water. One layer of sterile filter paper was placed at the base of each Petri dish (for *A. retroflexus* L., 12-well plates were used). Then, 3 mL (for *A. retroflexus* L., 330 μL) of the methanolic solution containing compounds were dropped on the filter paper. The final concentrations of tested compounds were 500, 250, 125, 62.5, and 31.25 μg/mL, respectively. The equivalent sterilized water was added to each well and Petri dish after the escape of methanol. Twenty-five (for *A. retroflexus* L., 10) viable seeds of weeds were placed on a filtrate paper. The herbicide glufosinate-ammonium was used as a positive control, and the methanol solution was then used as solvent control. Lids were sealed with Parafilm and incubated at 28°C, with 12 h supplemental light provided by 400 W Philips lamps and 26°C with 12 h darkness per day. The radicle and plumule lengths were measured and inhibition rates were calculated after 4 days. All treatments were carried out in triplicate. The experimental results are expressed as the mean ± SD, and the EC_50_ values were calculated from the regression equations.

The inhibition rate (expressed as a percentage) was calculated as follows:


$$\begin{array}{l} \frac{\text{radicle }\left(\text{plumule}\right)\text{length in the control}-\text{radicle }\left(\text{plumule}\right)\text{ length in the treatment}}{\text{radicle }\left(\text{plumule}\right)\text{ length in the control}}\; \end{array}$$

## Results and Discussion

### Structure Elucidation of the Isolated Compounds

**Figure 2:**
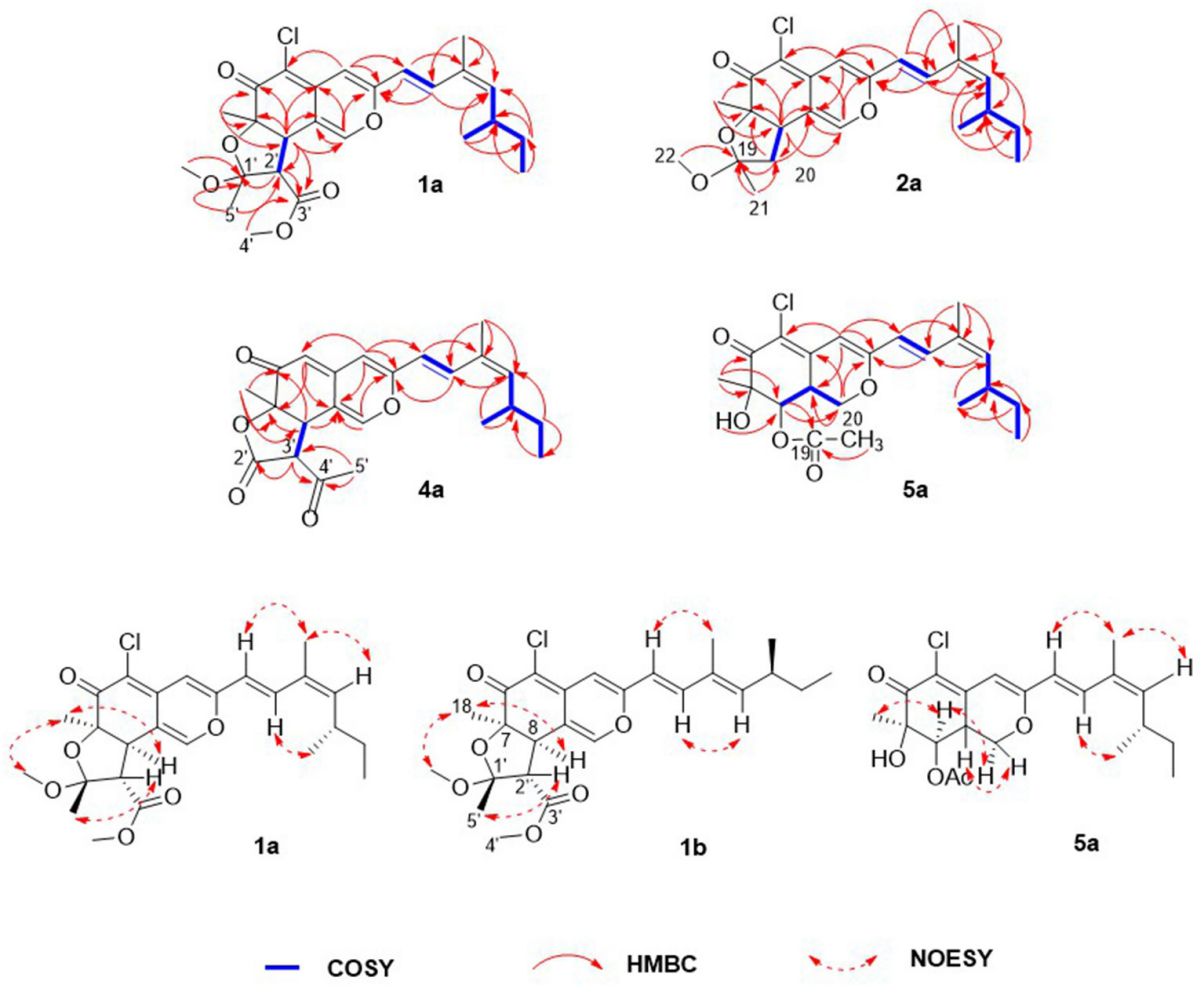
Selected key nuclear overhauser effect spectroscopy (NOESY), correlation spectroscopy (COSY), and heteronuclear multiple bond correlation (HMBC) correlations of **1a, 1b, 2a, 4a, and 5a**.

Compound **1** was isolated as a yellow, amorphous powder, and its molecular formula was deduced as C_25_H_31_O_6_Cl by HRESIMS (Supplementary Figure S7), corresponding to 10 degrees of unsaturation. The chlorine atom was confirmed by an isotopic peak for [M+H]^+^:[M+H+2]^+^ with an intensive ratio of 3:1 in the molecule. It existed as inseparable mixtures of two isomers according to HPLC analysis on either ODS or chiral column, due to spontaneous isomerization. The ^1^H and ^13^C NMR spectra (Tables 1, 2) of **1** showed two sets of resonances with a ratio of 1:4 for the **1a** and **1b** isomers. The ^1^H NMR spectroscopic data and heteronuclear singular quantum correlation (HSQC) correlations (Supplementary Figure S3) of **1a** revealed seven methyl groups, including five singlets (δ_H_/δ_C_ 1.45/24.6, 1.58/21.5, 1.89/20.1, 3.32/49.2, and 3.74/52.2), one doublet (δ_H_/δ_C_ 1.01/20.9), one triplet (δ_H_/δ_C_ 0.85/12), one methylene (δ_H_/δ_C_ 1.33/30.2, 1.44/30.2), three aliphatic methines (δ_H_/δ_C_ 2.64/34, 3.04/58.2, and 3.80/44.6), and five olefinic protons (δ_H_/δ_C_ 5.49/145.1, 6.14/118.8, 6.53/105.8, 7.41/133.4, and 7.56/146). Additionally, the ^13^C NMR spectra of **1a** revealed the presence of 25 carbons, including one conjugated ketone carbonyl at δ_C_ 188.4, one ester carbonyl at δ_C_ 169.3, one oxygenated quaternary olefinic carbon at δ_C_ 157.7, two sp^3^ oxygenated quaternary carbons signal at δ_C_ 83.6/105.6, and four quaternary olefinic carbons signal at δ_C_ 109.3/114.2/129.8/140.3. These NMR spectroscopic data indicated that **1a** belonged to the family of azaphilones and the planar structure was the same as that of isochromophilone C (Luo et al., 2018). Further examination found that the key nuclear overhauser effect spectroscopy (NOESY) correlations (Figure 2, Supplementary Figure S6) had obvious differences between **1a** and isochromophilone C, indicating they were diastereoisomers.

The relative configuration of **1a** was established by NOESY correlations (Figure 2, Supplementary Figure S6) and corresponding proton coupling constants (Luo et al., 2018; Qian et al., 2019). The large coupling constant (*J* = 12.6 Hz) between H-2' and H-8 suggested these two protons were on the opposite orientation. The NOESY correlations observed for H_3_-18, H-8, and 1'-OCH_3_ indicated that these protons were located on the same face. Thus, the stereochemistry of the azaphilone skeleton of **1a** was determined. Furthermore, the coupling constant between H-9 and H-10 (*J* = 15.6 Hz) in addition to the NOESY correlations between H-9/H-12 and 17-CH_3_, and between H-10 and 16-CH_3_ illustrated that the double bond at C-9 and C-10 was *E* configuration, and C-11/C-12 was *Z* configuration. Therefore, the relative configuration of **1a**, differing from that of isochromophilone C, was assigned as *rel*-(7*R*,8*R*,9*E*,11*Z*,1′*R*,2′*S*), and given the name as isochromophilone H.

The ^1^H and ^13^C NMR data of **1b** were similar to those of **1a**. The differences between them were the NMR data of C-9 to C-13, and C-17, indicating **1a**/**1b** were a pair of *E/Z* isomers, which was confirmed by the NOESY correlations of H-9/17-CH_3_, and H-10/H-12. Hence, **1b** was defined as *rel*-(7*R*,8*R*,9*E*,11*E*,1′*R*,2′*S*)-isochromophilone H.

Compounds **2**–**7** were also isolated as six pairs of C-11 *E/Z* isomers. Among them, **2a**, **3a**, **4a**, **5a**, and **6a** were new compounds with 11-(*Z*) configuration, which were confirmed by NOESY correlations of H-9/H-12 and 17-CH_3_, and H-10/16-CH_3_. The relative configuration of **5b** was also determined for the first time by the NOESY correlations (Supplementary Figure S34), combined with the proton coupling constants. The coupling constants calculated for H-8 (*J* = 10.2 Hz) and H-8a (*J* = 4.8, 10.2, and 13.2 Hz), indicated the ax/ax relationship of these two protons. The NOESY correlations observed for H-1α/H_3_-18 and H-8 suggested that these protons were cofacial. Accordingly, the relative configuration of **5b** was established as *rel*-(7*R*,8*R*,8a*R*).

**Figure 3:**
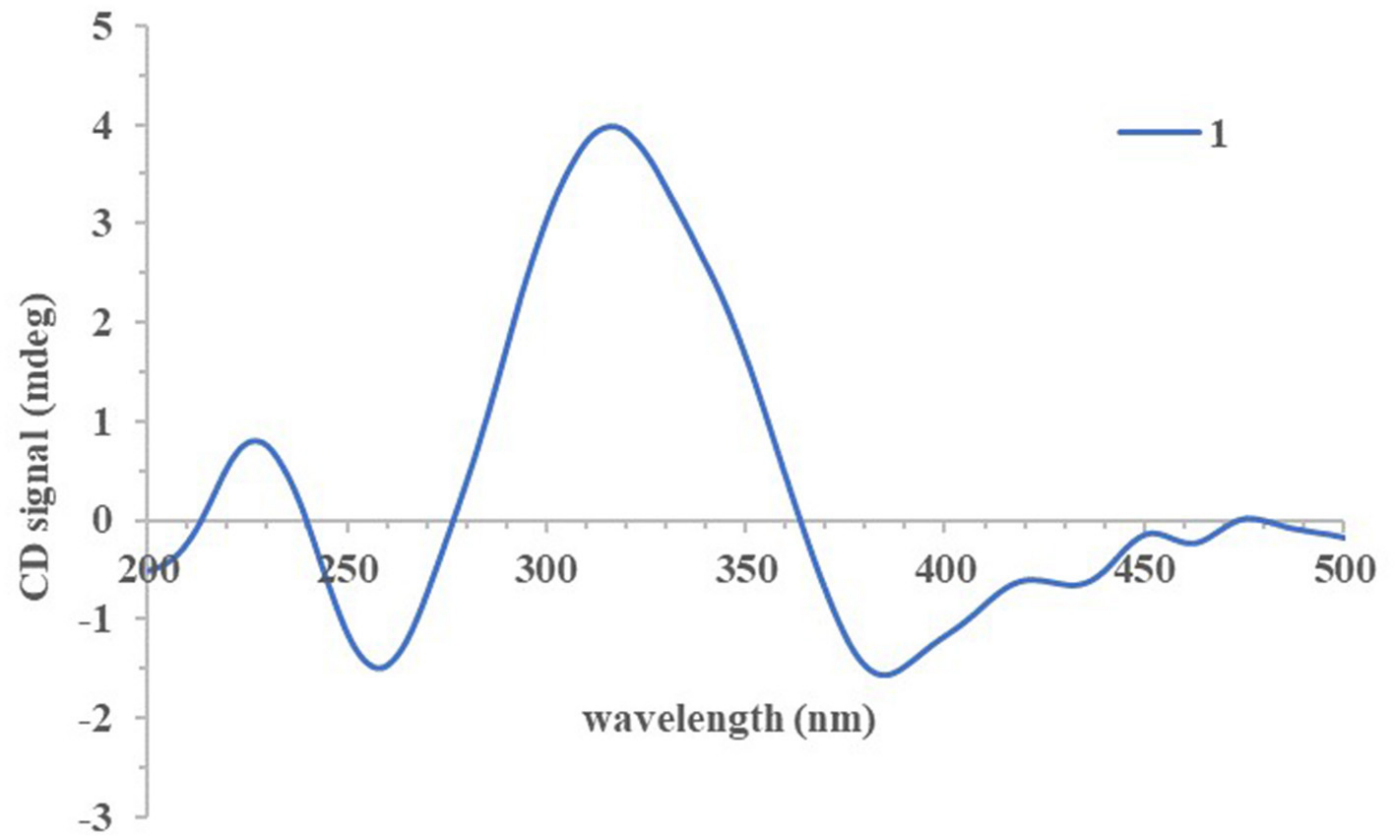
Experimental electronic circular dichroism (ECD) spectrum of compound **1**.

**Figure 4:**
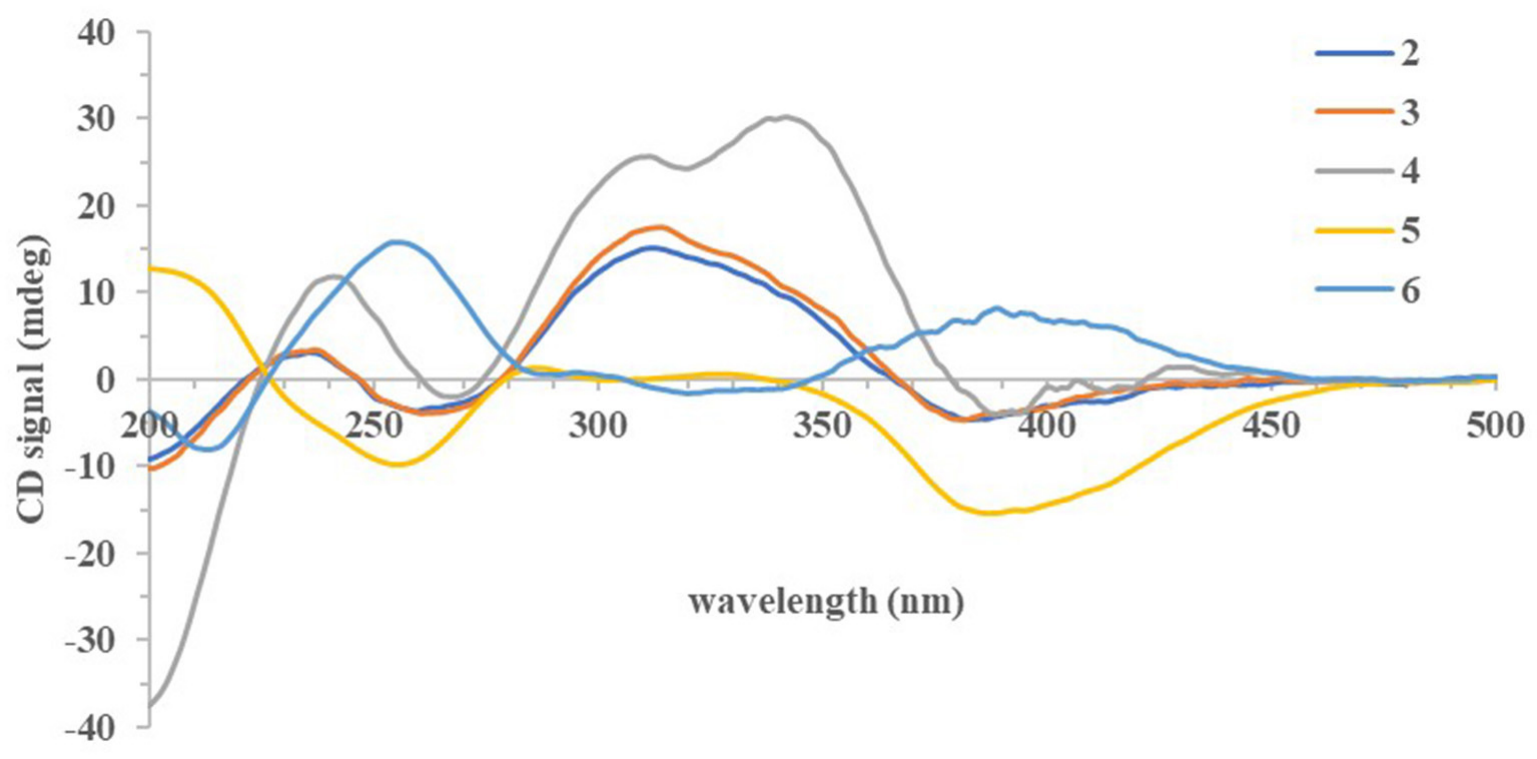
Experimental ECD spectra of compounds **2–6**.

The absolute configurations of all the isolated compounds were determined by comparison of experimental ECD spectra, and biosynthetic considerations. Among these compounds, the stereogenic carbon at C-13 in the side chain moiety was established to be *S* due to the aliphatic branch of this kind of azaphilones having a shared biosynthetic pathway (Gao et al., 2013). The absolute configuration of C-7 in compounds **1**–**5** was assigned to be *R* based on positive Cotton effects at 312 (Δε + 1.18, **1**), 312 (Δε + 3.82, **2**), 313 (Δε + 8.85, **3**), 310 (Δε + 6.11, **4**), and 325 nm (Δε + 0.32, **5**), respectively (Figures 3, 4; Qian et al., 2019). In addition, combined with the NOESY correlations, the absolute configurations of **1a** and **1b** were assigned as 7*R*,8*R*,13*S*,1′*R*,2′*S* (Luo et al., 2018). The absolute configurations of **2**, **3**, **4**, and **5** were suggested to be (7*R*,8*R*,19*S*), (7*R*,8*R*,19*R*), (7*R*,8*R*,3′*R*), and (7*R*,8*R*,8a*R*) by comparisons of experimental ECD data to those reported in the literature (Figure 4; Arai et al., 1995; Matsuzaki et al., 1995; Qian et al., 2019). Contrary to ECD data of **5** and *epi*-isochromophilone III, the ECD spectrum (Figure 4) in MeOH of **6** exhibited Cotton effects at 388 (Δε +1.88), 321 (Δε −0.34), 255 (Δε +3.47), and 214 nm (Δε −1.87), indicating the absolute configuration at C-7 of **6** was *S*. Thus, **6** was identified as a new compound, and the absolute configurations of **6a** and **6b** were confirmed as 7*S*,8*R*,8a*S*,13*S* (Hemtasin et al., 2016). Compounds **2b**–**5b** were identified as the isomers of **2a**–**5a** with 11-(*E*) configuration reported in the literature (Arai et al., 1995; Matsuzaki et al., 1995; Pairet et al., 1995; Qian et al., 2019). Compounds **7a** and **7b** were identified as isochromophilone Ib and Ia by comparing their NMR data with those in the literature (Omura et al., 1993; Matsuzaki et al., 1995).

### Phytotoxicity Bioassays

Numerous studies have reported that azaphilones have broad-spectrum biological activities. However, there were relatively few reports about their phytotoxic activity of them. Chaetomugilin A, D, S, and O, four chlorine-containing azaphilone derivatives, isolated from the endophytic *Chaetomium globosum* TY1, showed a higher response index and lower IC_50_ values to eight species of herbaceous plant seeds than positive control glyphosate (Wang et al., 2017). Chaetomugilin D and J, isolated from the EtOAc extract of the fermentation medium of *C. globosum*, exhibited phytotoxicity to lettuce seeds, with IC_50_ values for root inhibition of 24.2 and 22.6 ppm, respectively, while those for shoot inhibition were 27.8 and 21.9 ppm, respectively (Piyasena et al., 2015). Acetosellin, isolated from large-scale cultures of the fungus *Cercosporella acetosella*, inhibited the growth of the root of *Lepidium sativum* and *Zea mais* at 640 μM (Gianluca et al., 2002). In the present work, all the isolated compounds were evaluated for their phytotoxicity against four weeds species (*A. fatua* L., *L. perenne* L., *A. retroflexus* L., *A. theophrasti* Medikus) in farmland. The experimental results indicated that sclerotiorin B (**3**), ochlephilone (**4**), and isochromophilone I (**7**) exhibited potent phytotoxicity against the growth of radicle and plumule of *A. retroflexus* L., with EC_50_ values ranging from 234.87 to 320.84 μM, compared to positive control glufosinate-ammonium (Table 3). Compounds **4** and **7** also showed inhibitory activities against the growth of velvetleaf (*A. theophrasti* Medikus; Table 4). These tested compounds had no significant inhibitory effects on the growth and germination of wild oat (*A. fatua* L.) and ryegrass (*L. perenne* L.).

**Table 3:** EC_50_ values of compounds **3**, **4**, and **7** in Redroot Amaranth.

**Compound**	**EC**_**50**_ **(*****μ*****M)**	
	**Plumule**	**Radicle**
**3**	320.84	271.48
**4**	287.07	234.87
**7**	288.36	240.30
Glufosinate ammonium*^a^*	656.04	555.11

a *Positive control*.

**Table 4:** EC_50_ Values of Compounds **4** and **7** in Velvetleaf.

**Compound**	**EC**_**50**_ **(*****μ*****M)**	
	**Plumule**	**Radicle**
**4**	939.49	1122.17
**7**	768.97	1201.52
Glufosinate ammonium*^a^*	555.11	807.43

a *Positive control*.

## Conclusions

In conclusion, we described seven pairs of azaphilones 11-(*E/Z*) isomers, including eight new compounds. Their structures and absolute configurations were elucidated based on comprehensive spectroscopic analysis and the comparisons of ECD data. Sclerotiorin B (**3**), ochlephilone (**4**), and isochromophilone I (**7**) exhibited potent phytotoxicity toward the growth of radicle and plumule of *A. retroflexus* L., compared to glufosinate-ammonium. This will provide new leading compounds for the research and development of marine-derived bioherbicides.

## Data Availability Statement

The original contributions presented in the study are included in the article/Supplementary Material, further inquiries can be directed to the corresponding author/s.

## Author Contributions

G-XW and C-SZ conceived and designed the experiments. WW and MW performed the experiments. WW, D-LZ, X-BW, J-LD, Y-QL, M-XL, and XG analyzed the experimental data. WW wrote the manuscript. D-LZ revised the article. All authors contributed to the article, reviewed the manuscript, and approved the submitted version.

## Funding

This work was financially supported by Reserve Talents for Yunnan Young and Middle-aged Academic and Technical Leaders (No.202105AC160037), the National Natural Science Foundation of China (41806194), the Agricultural Science and Technology Project of Guizhou Province (2021XM10 and 201803), and the Fundamental Research Funds for Central Non-profit Scientific Institution (1610232021007).

## Conflict of Interest

X-BW and J-LD are employed by Guizhou Tobacco Company. The remaining authors declare that the research was conducted in the absence of any commercial or financial relationships that could be construed as a potential conflict of interest.

## Publisher's Note

All claims expressed in this article are solely those of the authors and do not necessarily represent those of their affiliated organizations, or those of the publisher, the editors and the reviewers. Any product that may be evaluated in this article, or claim that may be made by its manufacturer, is not guaranteed or endorsed by the publisher.
