# Supplementary material for: Phytotoxic Azaphilones From the Mangrove-Derived Fungus Penicillium sclerotiorum HY5
Source: Front Microbiol. 2022 Apr 19;13:880874. doi: 10.3389/fmicb.2022.880874 (PMC9063783; doi:10.3389/fmicb.2022.880874)
Supplement: Supplementary file 1 [file Table_1.DOCX]

***Supporting Information***

**Phytotoxic Azaphilones from the Mangrove-Derived Fungus *Penicillium sclerotiorum* HY5**

**Wei Wang^1^, Mei Wang^2^, Xian-Bo Wang^3^, Yi-Qiang Li^2^, Ji-Lin Ding^3^, Ming-Xian Lan^1^, Xi Gao^1^, Dong-Lin Zhao^2^*, Cheng-Sheng Zhang^2^*, and Guo-Xing Wu^1^***

^1^College of Plant Protection, Yunnan Agricultural University, Kunming 650201, People’s Republic of China

^2^Tobacco Research Institute of Chinese Academy of Agricultural Sciences, Qingdao 266101, People’s Republic of China

^3^Zunyi Branch, Guizhou Tobacco Company, Zunyi 563000, People’s Republic of China

***Correspondence:**Dong-Lin Zhao
zhaodonglin@caas.cn

Cheng-Sheng Zhang
zhchengsheng@126.com

Guo-Xing Wu

wugx1@163.com

**Keywords: Azaphilones, Phytotoxicity, Bioherbicide, Weeds, *Penicillium sclerotiorum***

**List of Supporting Information**

**Figure S1.** ^1^H NMR (600 MHz, chloroform-*d*) spectrum of compound **1**

**Figure S2.** ^13^C NMR (150 MHz, chloroform-*d*) spectrum of compound **1**

**Figure S3.** HSQC (chloroform-*d*) spectrum of compound **1**

**Figure S4.** ^1^H-^1^H COSY (chloroform-*d*) spectrum of compound **1**

**Figure S5.** HMBC (chloroform-*d*) spectrum of compound **1**

**Figure S6.** NOESY (chloroform-*d*) spectrum of compound **1**

**Figure S7.** HRESIMS spectrum of compound **1**

**Figure S8.** ^1^H NMR (500 MHz, chloroform-*d*) spectrum of compound **2**

**Figure S9.** ^13^C NMR (125 MHz, chloroform-*d*) spectrum of compound **2**

**Figure S10.** HSQC (chloroform-*d*) spectrum of compound **2**

**Figure S11.** ^1^H-^1^H COSY (chloroform-*d*) spectrum of compound **2**

**Figure S12.** HMBC (chloroform-*d*) spectrum of compound **2**

**Figure S13.** NOESY (chloroform-*d*) spectrum of compound **2**

**Figure S14.** HRESIMS spectrum of compound **2**

**Figure S15.** ^1^H NMR (500 MHz, chloroform-*d*) spectrum of compound **3**

**Figure S16.** ^13^C NMR (125 MHz, chloroform-*d*) spectrum of compound **3**

**Figure S17.** HSQC (chloroform-*d*) spectrum of compound **3**

**Figure S18.** ^1^H-^1^H COSY (chloroform-*d*) spectrum of compound **3**

**Figure S19.** HMBC (chloroform-*d*) spectrum of compound **3**

**Figure S20.** NOESY (chloroform-*d*) spectrum of compound **3**

**Figure S21.** HRESIMS spectrum of compound **3**

**Figure S22.** ^1^H NMR (500 MHz, chloroform-*d*) spectrum of compound **4**

**Figure S23.** ^13^C NMR (125 MHz, chloroform-*d*) spectrum of compound **4**

**Figure S24.** HSQC (chloroform-*d*) spectrum of compound **4**

**Figure S25.** ^1^H-^1^H COSY (chloroform-*d*) spectrum of compound **4**

**Figure S26.** HMBC (chloroform-*d*) spectrum of compound **4**

**Figure S27.** NOESY (chloroform-*d*) spectrum of compound **4**

**Figure S28.** HRESIMS spectrum of compound **4**

**Figure S29.** ^1^H NMR (600 MHz, chloroform-*d*) spectrum of compound **5**

**Figure S30.** ^13^C NMR (150 MHz, chloroform-*d*) spectrum of compound **5**

**Figure S31.** HSQC (chloroform-*d*) spectrum of compound **5**

**Figure S32.** ^1^H-^1^H COSY (chloroform-*d*) spectrum of compound **5**

**Figure S33.** HMBC (chloroform-*d*) spectrum of compound **5**

**Figure S34.** NOESY (chloroform-*d*) spectrum of compound **5**

**Figure S35.** HRESIMS spectrum of compound **5**

**Figure S36.** ^1^H NMR (600 MHz, chloroform-*d*) spectrum of compound **6**

**Figure S37.** ^13^C NMR (150 MHz, chloroform-*d*) spectrum of compound **6**

**Figure S38.** HSQC (chloroform-*d*) spectrum of compound **6**

**Figure S39.** ^1^H-^1^H COSY (chloroform-*d*) spectrum of compound **6**

**Figure S40.** HMBC (chloroform-*d*) spectrum of compound **6**

**Figure S41.** NOESY (chloroform-*d*) spectrum of compound **6**

**Figure S42.** HRESIMS spectrum of compound **6**

**Figure S43** Regression equation of inhibition rate of compound **3** against the growth of *A. retroflexus* L.

**Figure S44** Regression equation of inhibition rate of compound **4** against the growth of *A. retroflexus* L.

**Figure S45** Regression equation of inhibition rate of compound **7** against the growth of *A. retroflexus* L.

**Figure S46** Regression equation of inhibition rate of glufosinate ammonium against the growth of *A. retroflexus* L.

**Table S47** The germination effect of *A. retroflexus* L. seeds inhibited by different concentrations of compound **3**

**Table S48** The germination effect of *A. retroflexus* L. seeds inhibited by different concentrations of compound **4**

**Table S49** The germination effect of *A. retroflexus* L. seeds inhibited by different concentrations of compound **7**

**Table S50** The germination effect of *A. retroflexus* L. seeds inhibited by different concentrations of glufosinate ammonium

**Figure S51** Regression equation of inhibition rate of compound **4** against the growth of *Abutilon theophrasti* Medikus

**Figure S52** Regression equation of inhibition rate of compound **7** against the growth of *Abutilon theophrasti* Medikus

**Figure S53** Regression equation of inhibition rate of glufosinate ammonium against the growth of *Abutilon theophrasti* Medikus

**Table S54** The germination effect of *Abutilon theophrasti* Medikus seeds inhibited by different concentrations of compound **4**

**Table S55** The germination effect of *Abutilon theophrasti* Medikus seeds inhibited by different concentrations of compound **7**

**Table S56** The germination effect of *Abutilon theophrasti* Medikus seeds inhibited by different concentrations of glufosinate ammonium


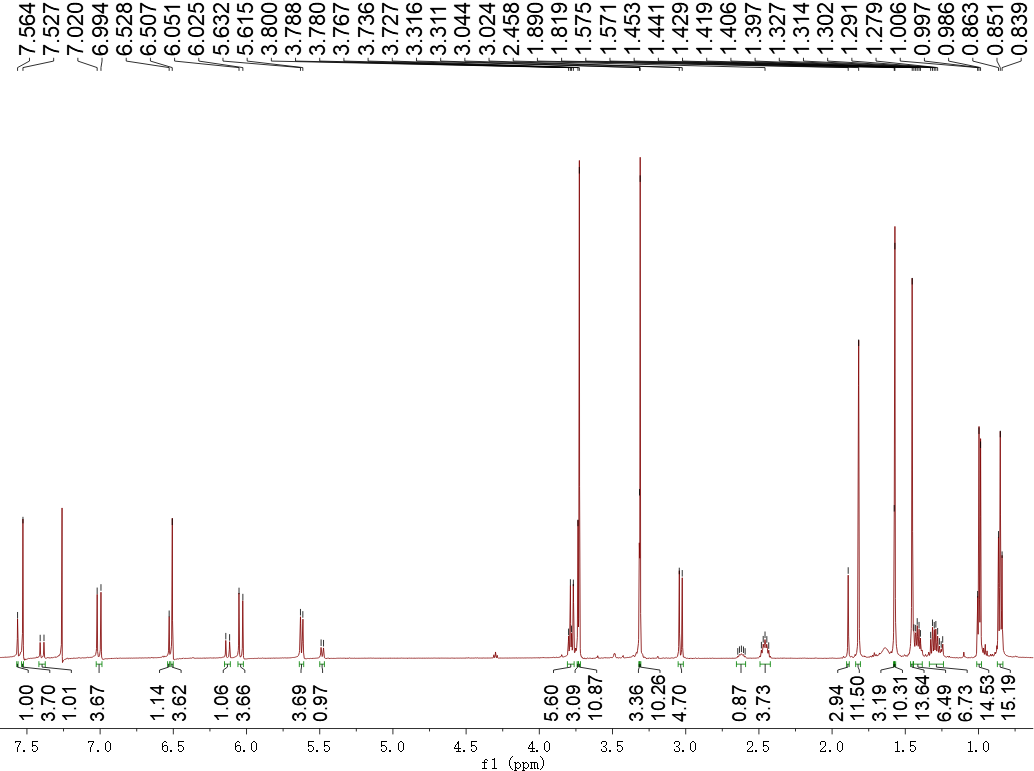


**Figure S1.** ^1^H NMR (600 MHz, chloroform-*d*) spectrum of compound **1**

**
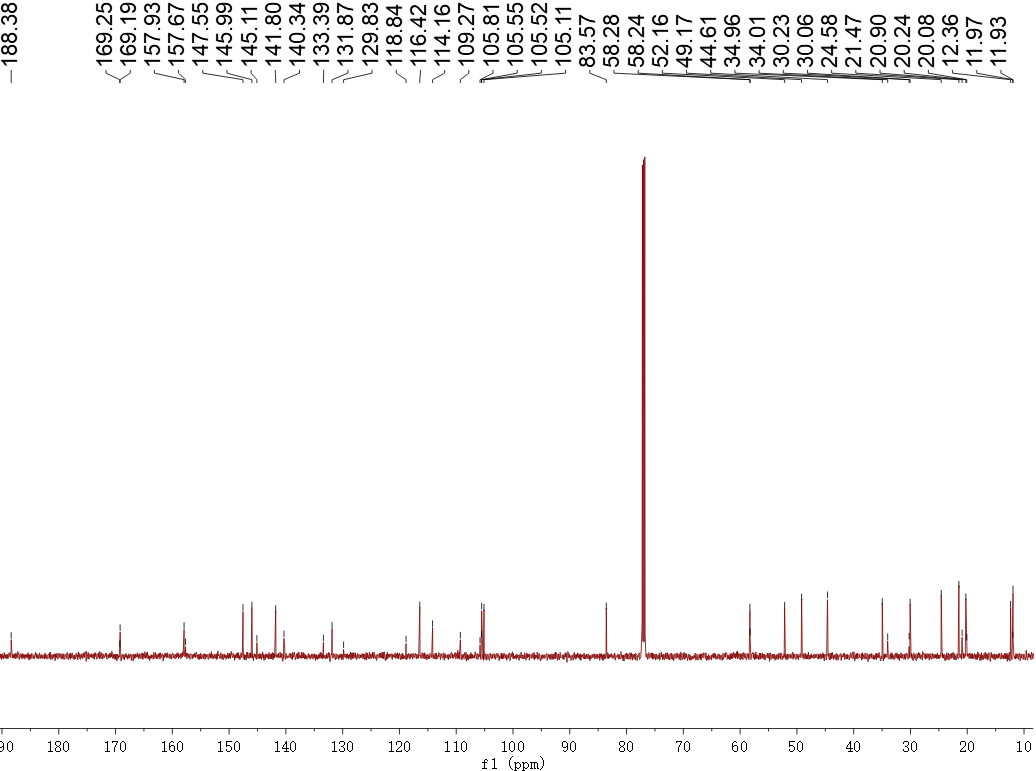
**

**Figure S2.** ^13^C NMR (150 MHz, chloroform-*d*) spectrum of compound **1**


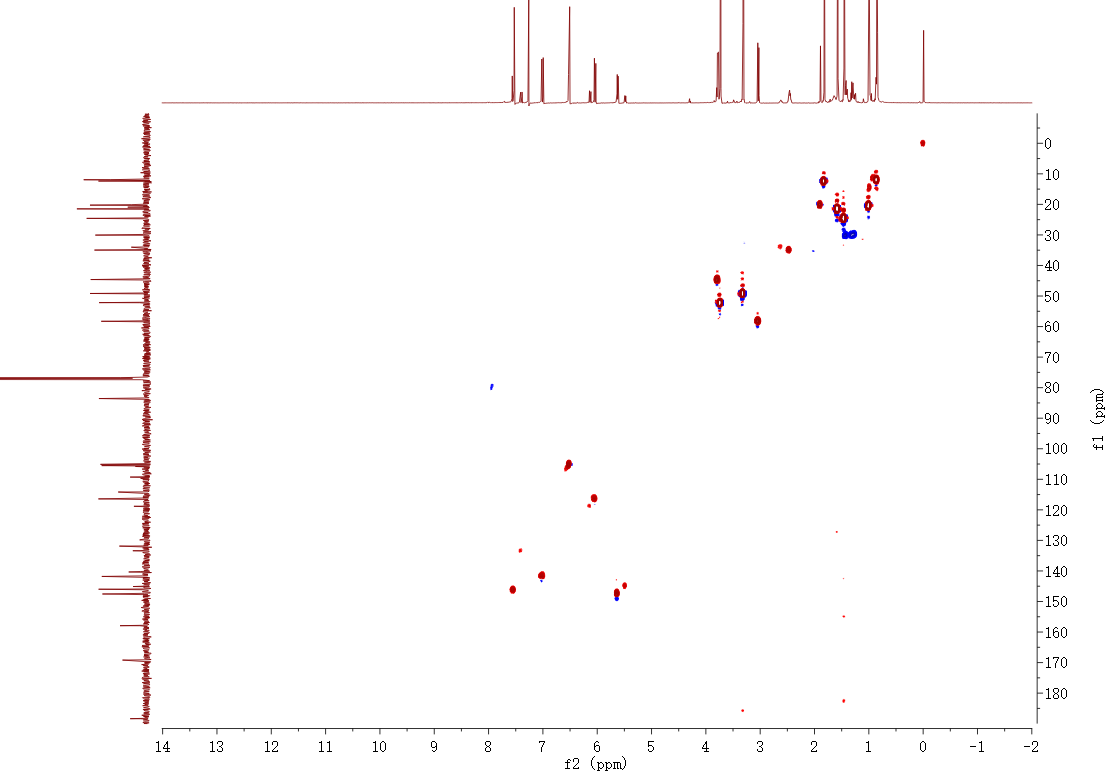


**Figure S3.** HSQC (chloroform-*d*) spectrum of compound **1**


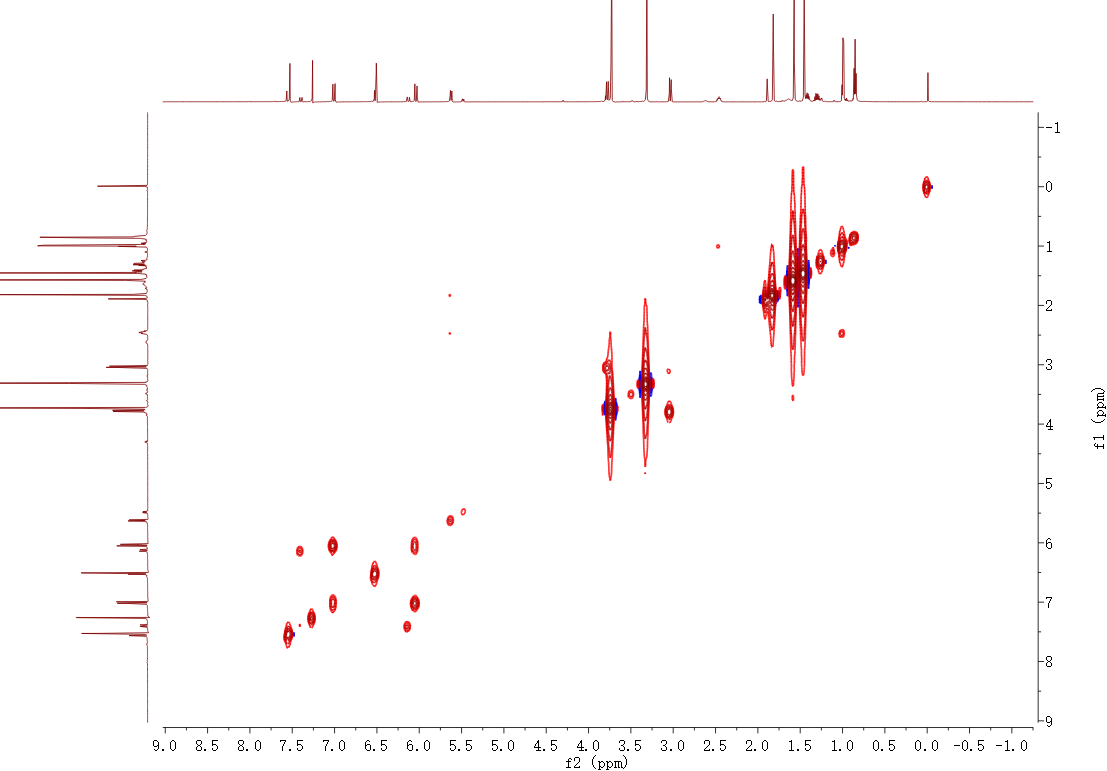


**Figure S4.** ^1^H-^1^H COSY (chloroform-*d*) spectrum of compound **1**


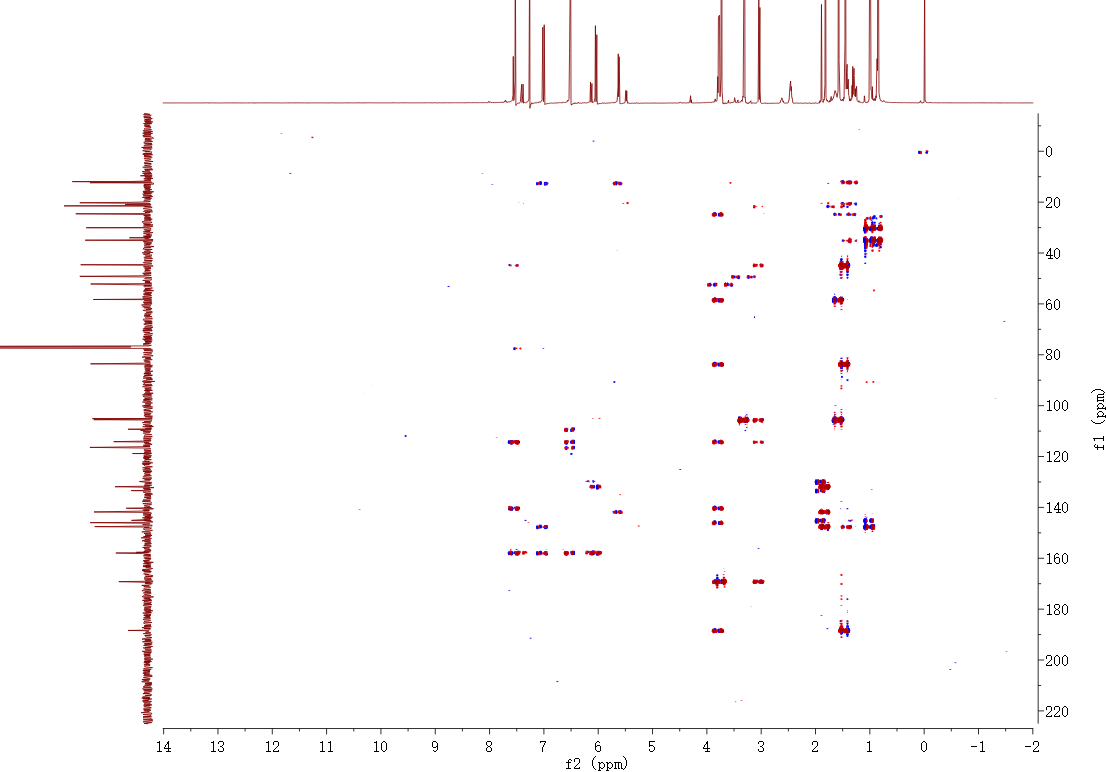


**Figure S5.** HMBC (chloroform-*d*) spectrum of compound **1**


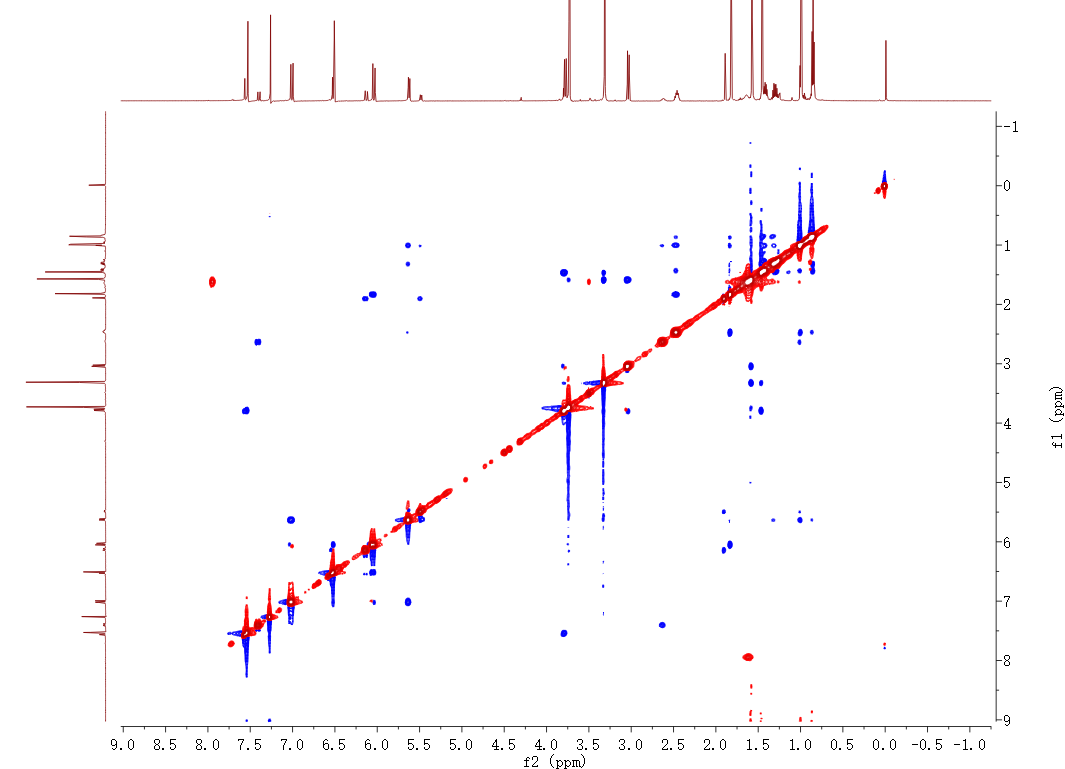


**Figure S6.** NOESY (chloroform-*d*) spectrum of compound **1**

**Figure S7.** HRESIMS spectrum of compound **1**

**
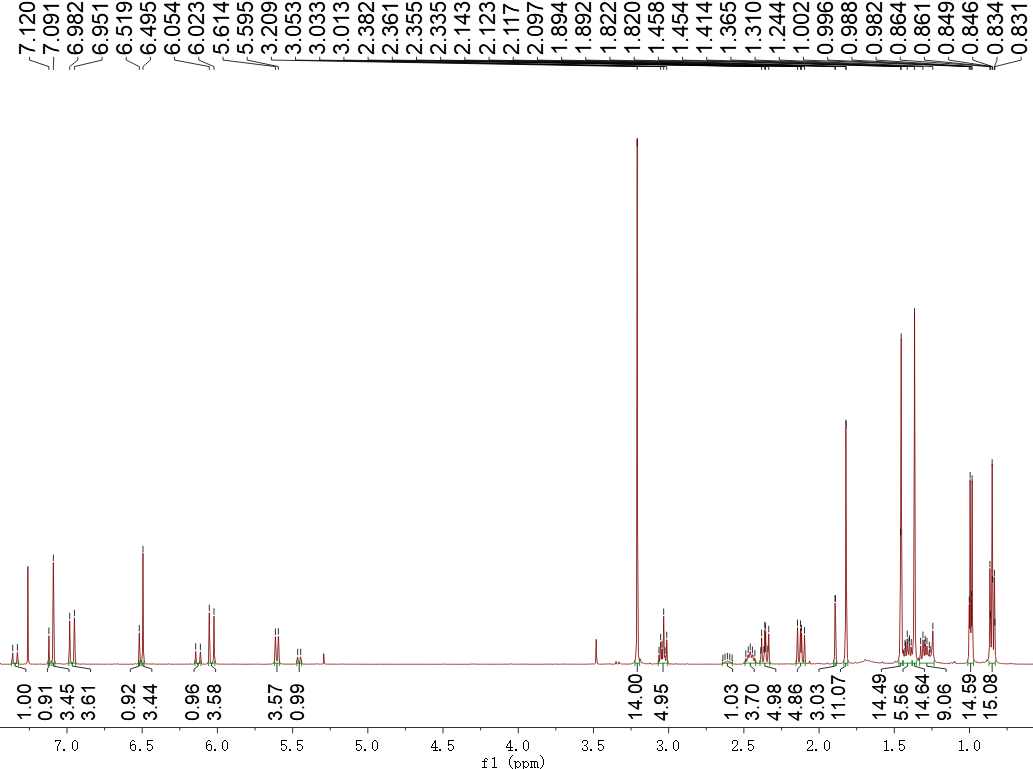
**

**Figure S8.** ^1^H NMR (500 MHz, chloroform-*d*) spectrum of compound **2**

**
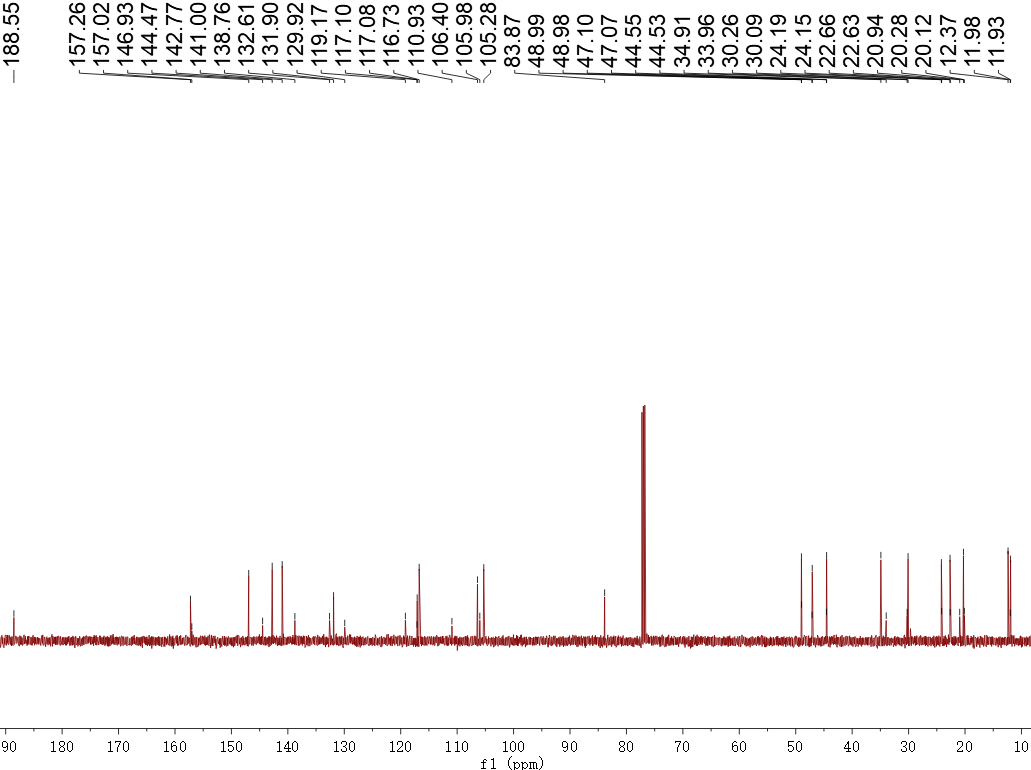
**

**Figure S9.** ^13^C NMR (125 MHz, chloroform-*d*) spectrum of compound **2**


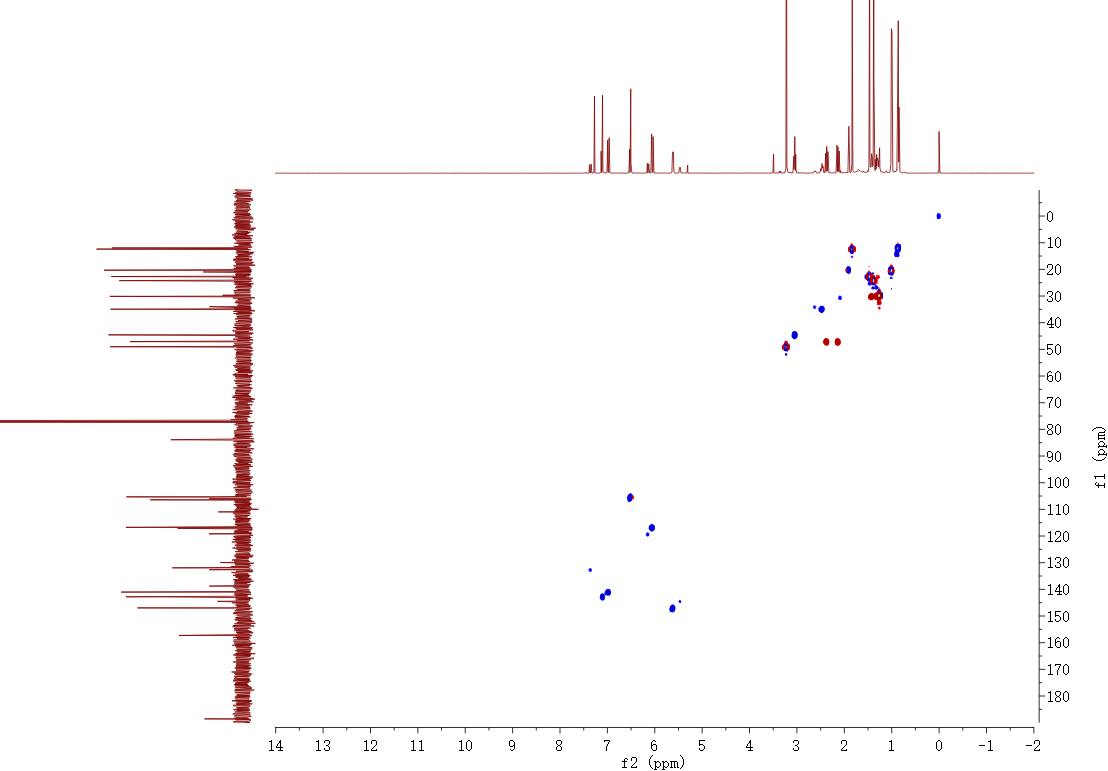


**Figure S10.** HSQC (chloroform-*d*) spectrum of compound **2**


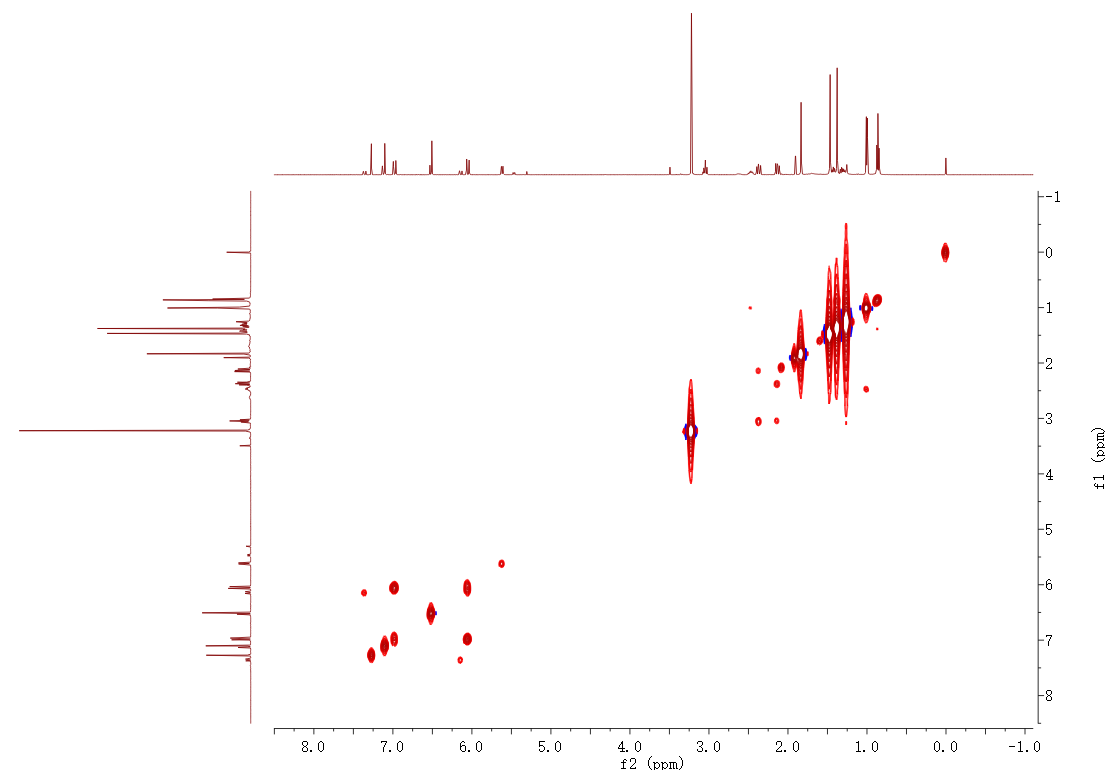


**Figure S11.** ^1^H-^1^H COSY (chloroform-*d*) spectrum of compound **2**


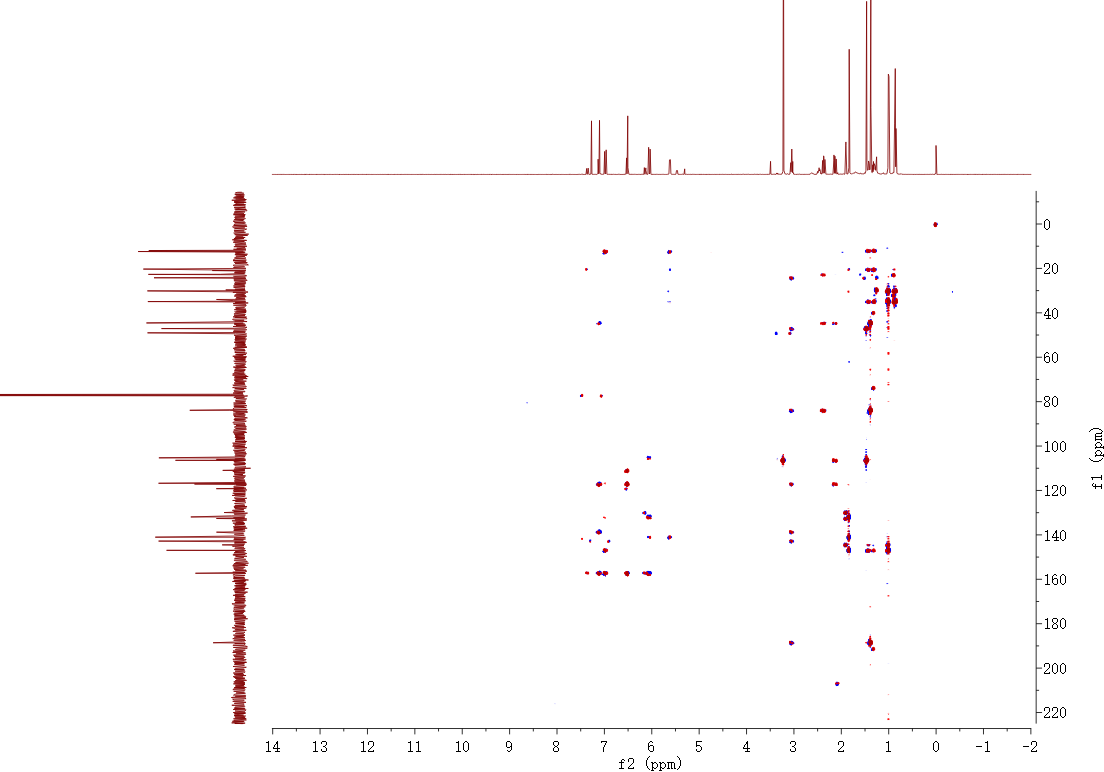


**Figure S12.** HMBC (chloroform-*d*) spectrum of compound **2**

**
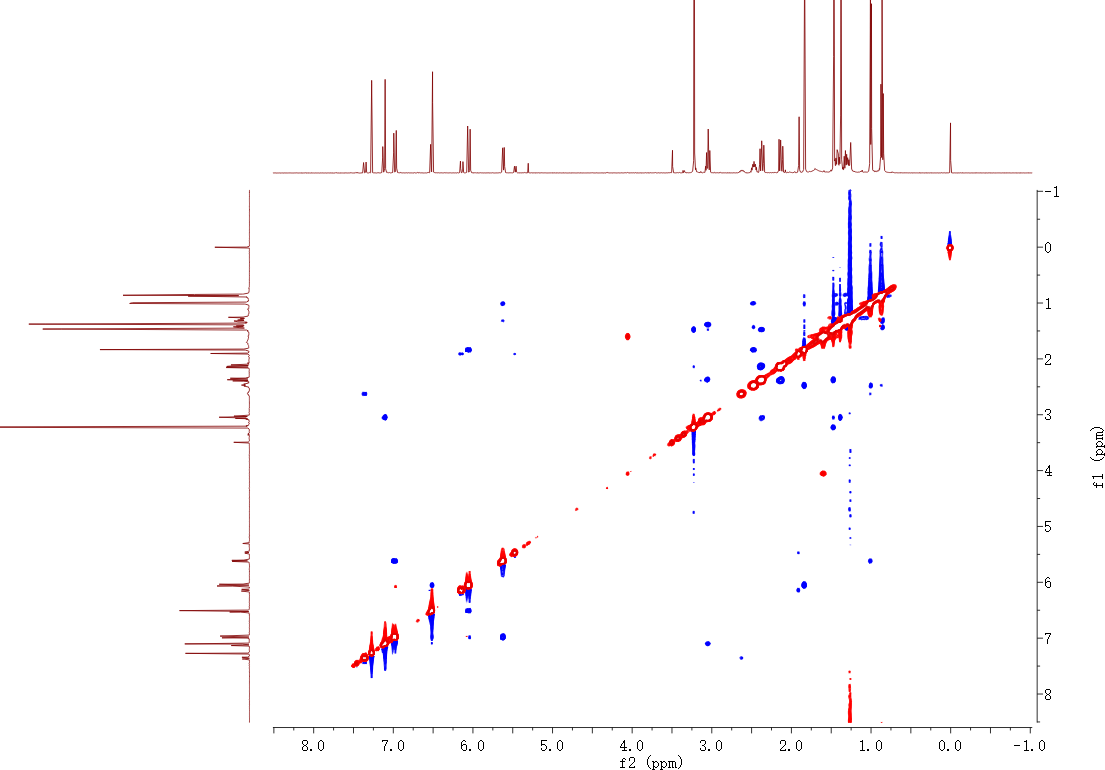
**

**Figure S13.** NOESY (chloroform-*d*) spectrum of compound **2**

**Figure S14.** HRESIMS spectrum of compound **2**

**
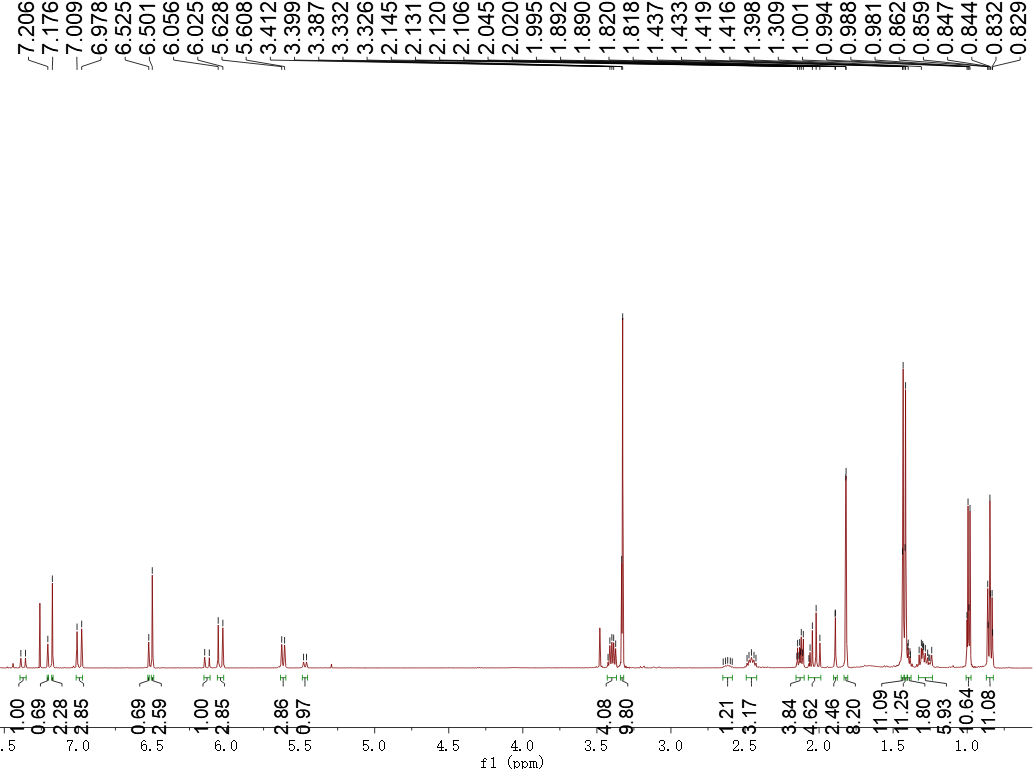
**

**Figure S15.** ^1^H NMR (500 MHz, chloroform-*d*) spectrum of compound **3**

**
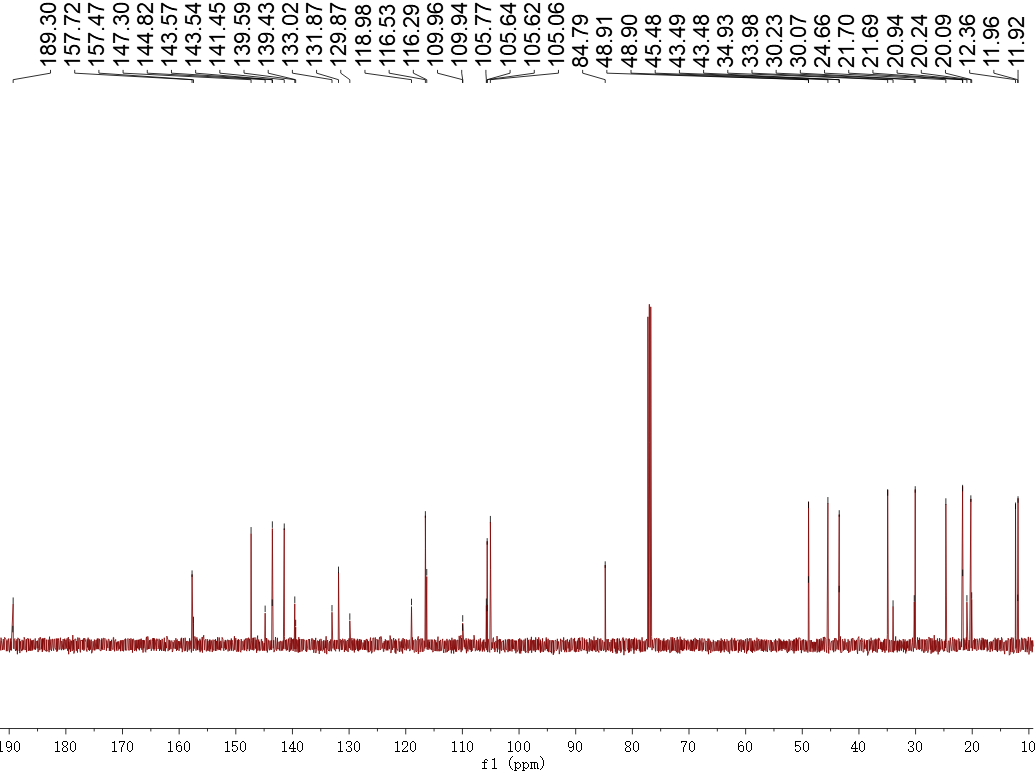
**

**Figure S16.** ^13^C NMR (125 MHz, chloroform-*d*) spectrum of compound **3**

**
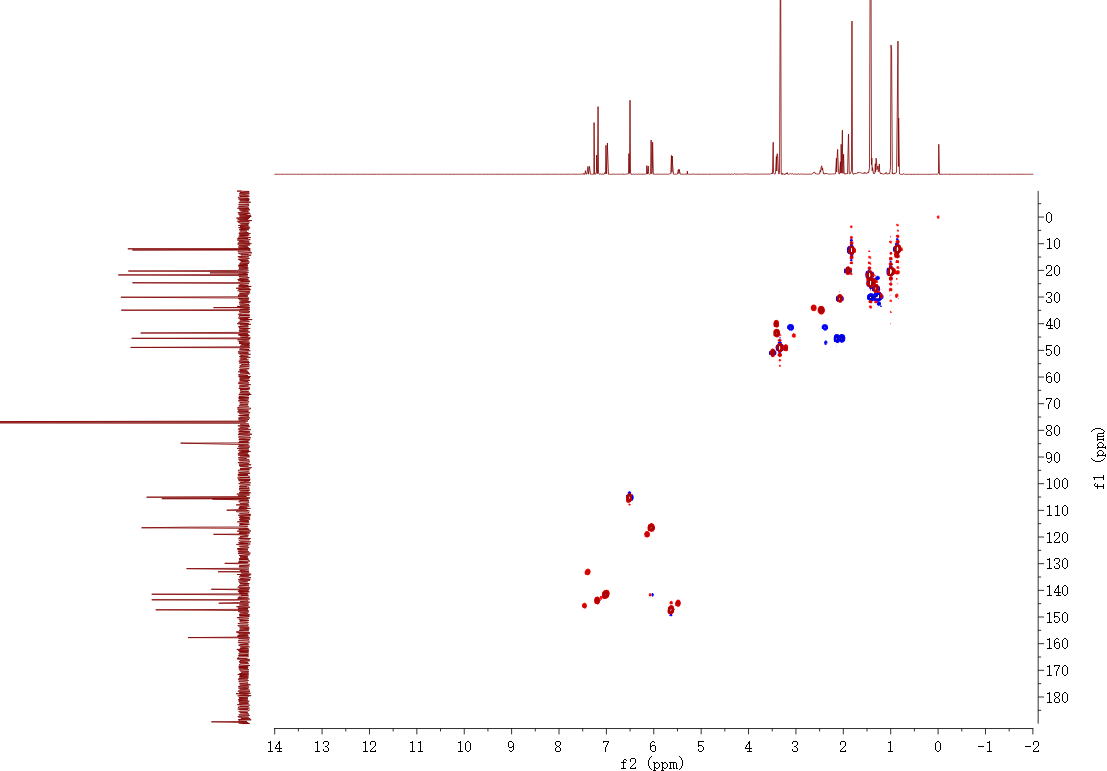
**

**Figure S17.** HSQC (chloroform-*d*) spectrum of compound **3**

**
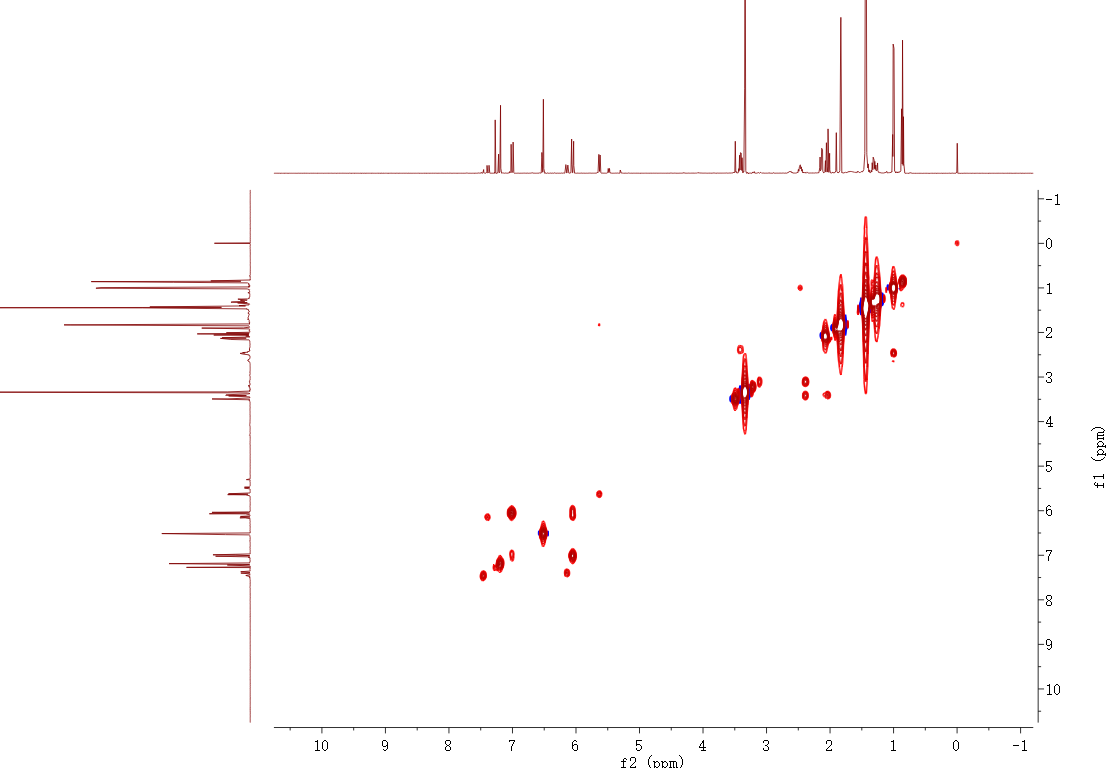
**

**Figure S18.** ^1^H-^1^H COSY (chloroform-*d*) spectrum of compound **3**

**
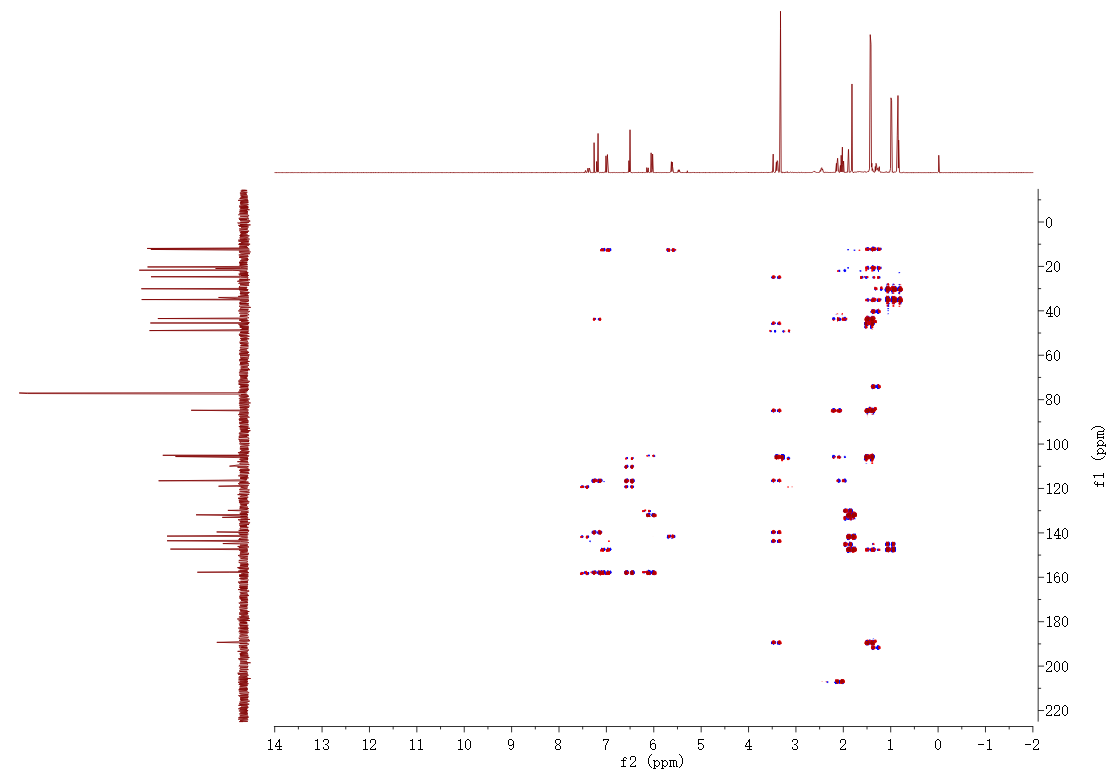
**

**Figure S19.** HMBC (chloroform-*d*) spectrum of compound **3**

**
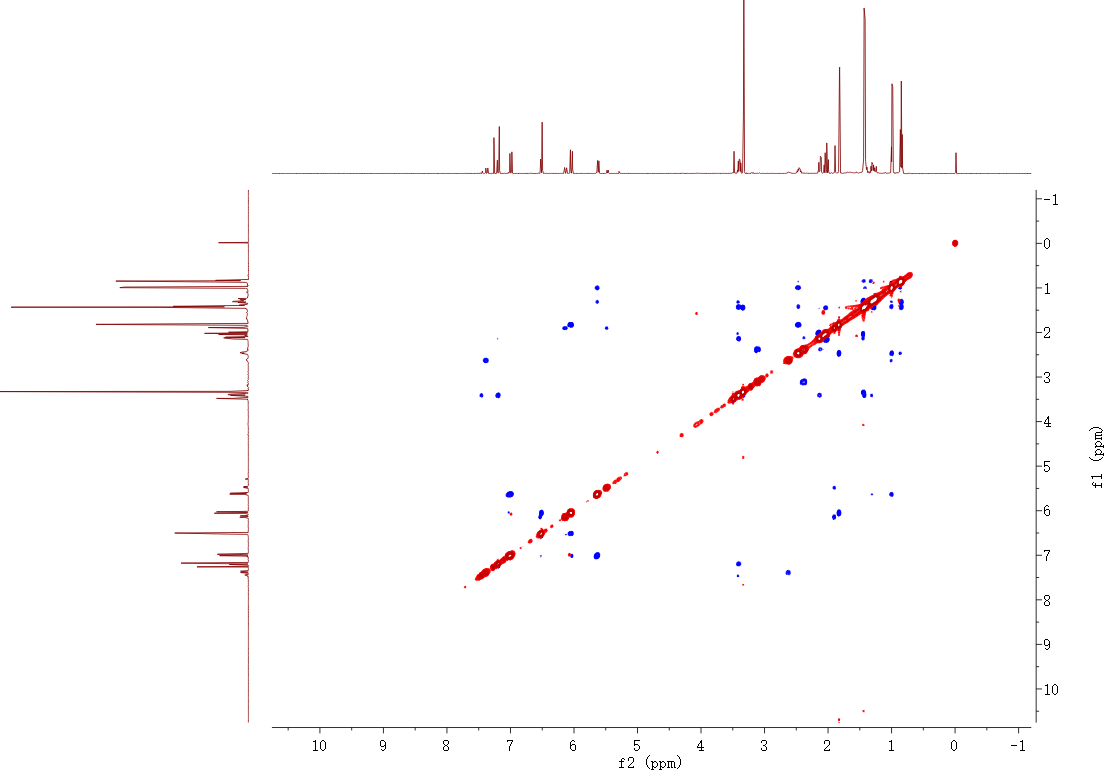
**

**Figure S20.** NOESY (chloroform-*d*) spectrum of compound **3**

**Figure S21.** HRESIMS spectrum of compound **3**

**
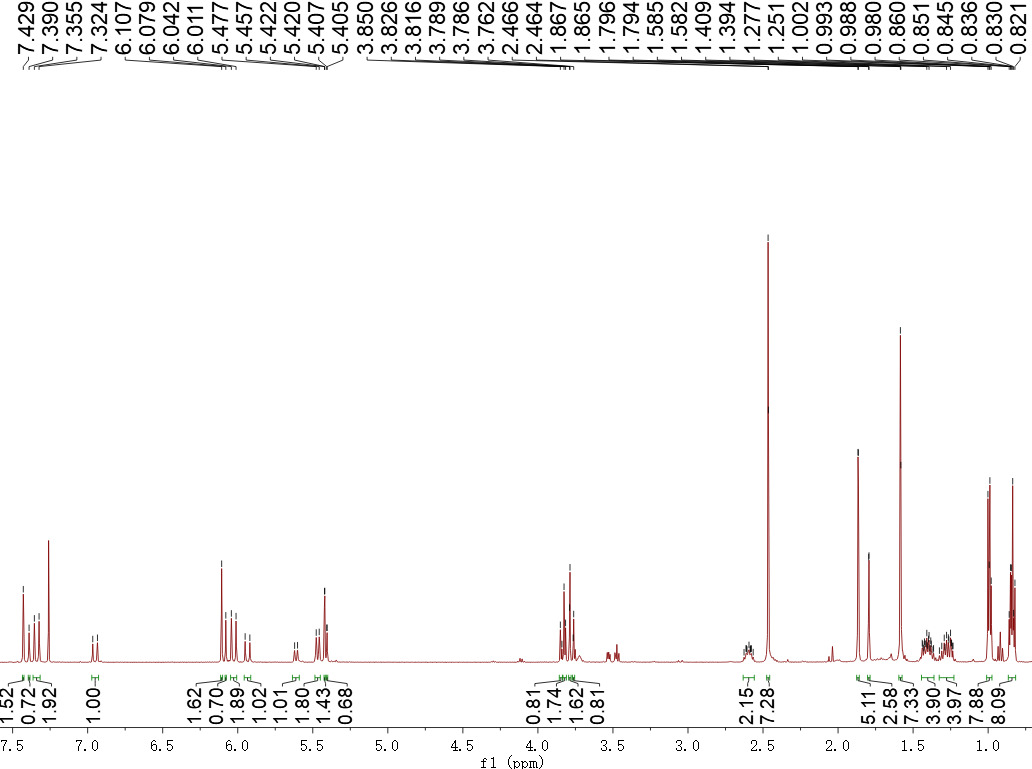
**

**Figure S22.** ^1^H NMR (500 MHz, chloroform-*d*) spectrum of compound **4**

**
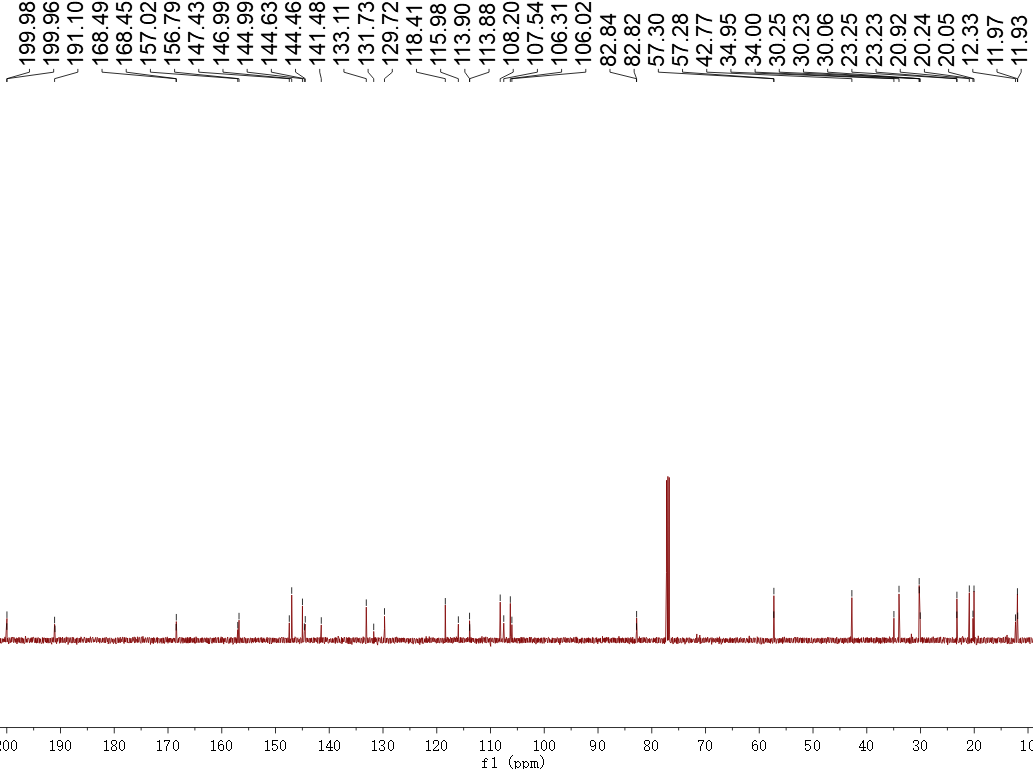
**

**Figure S23.** ^13^C NMR (125 MHz, chloroform-*d*) spectrum of compound **4**

**
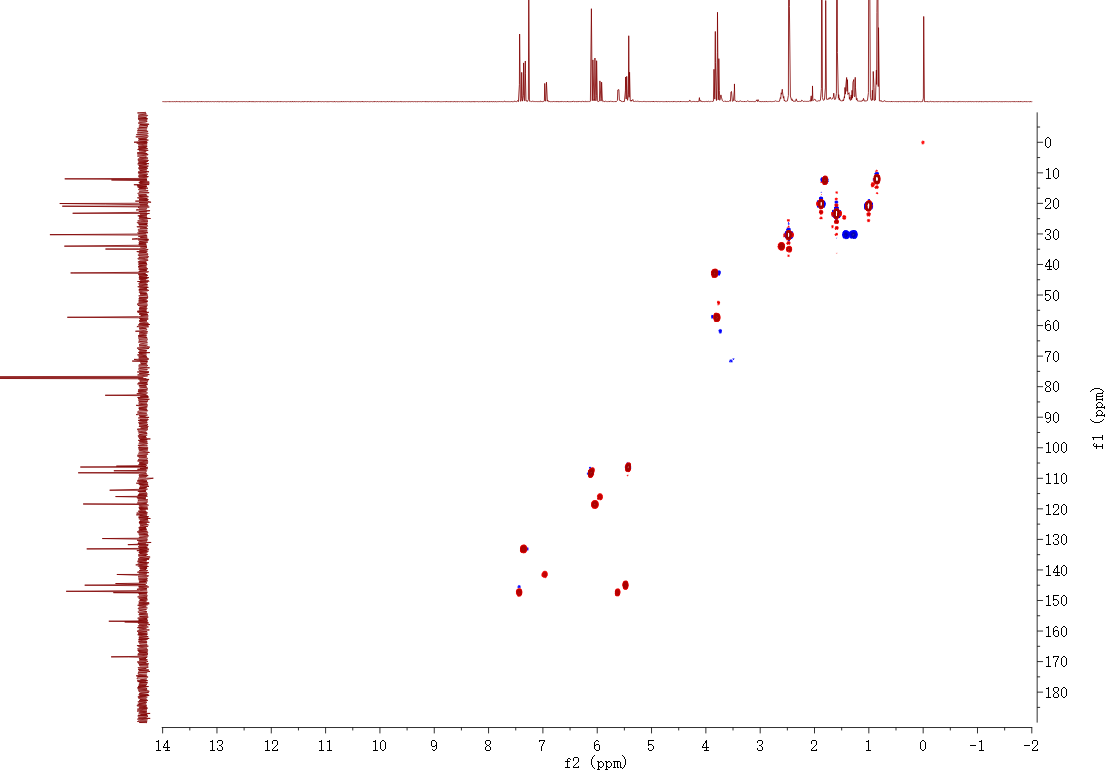
**

**Figure S24.** HSQC (chloroform-*d*) spectrum of compound **4**

**
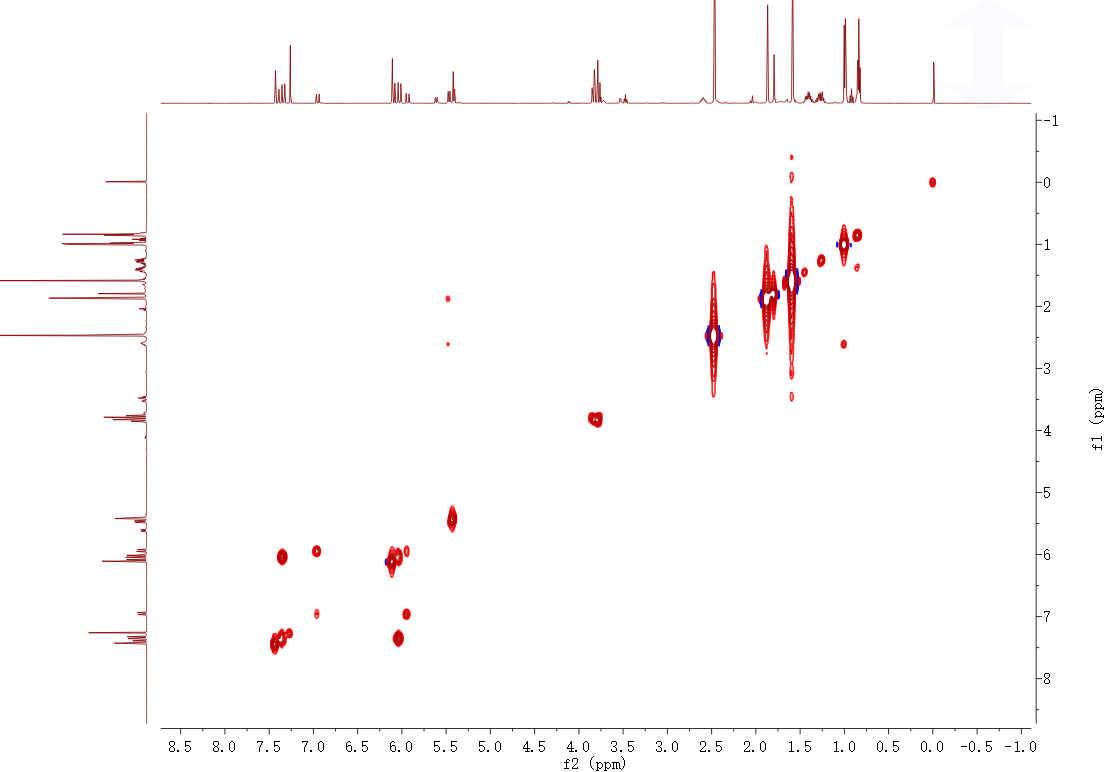
**

**Figure S25.** ^1^H-^1^H COSY (chloroform-*d*) spectrum of compound **4**

**
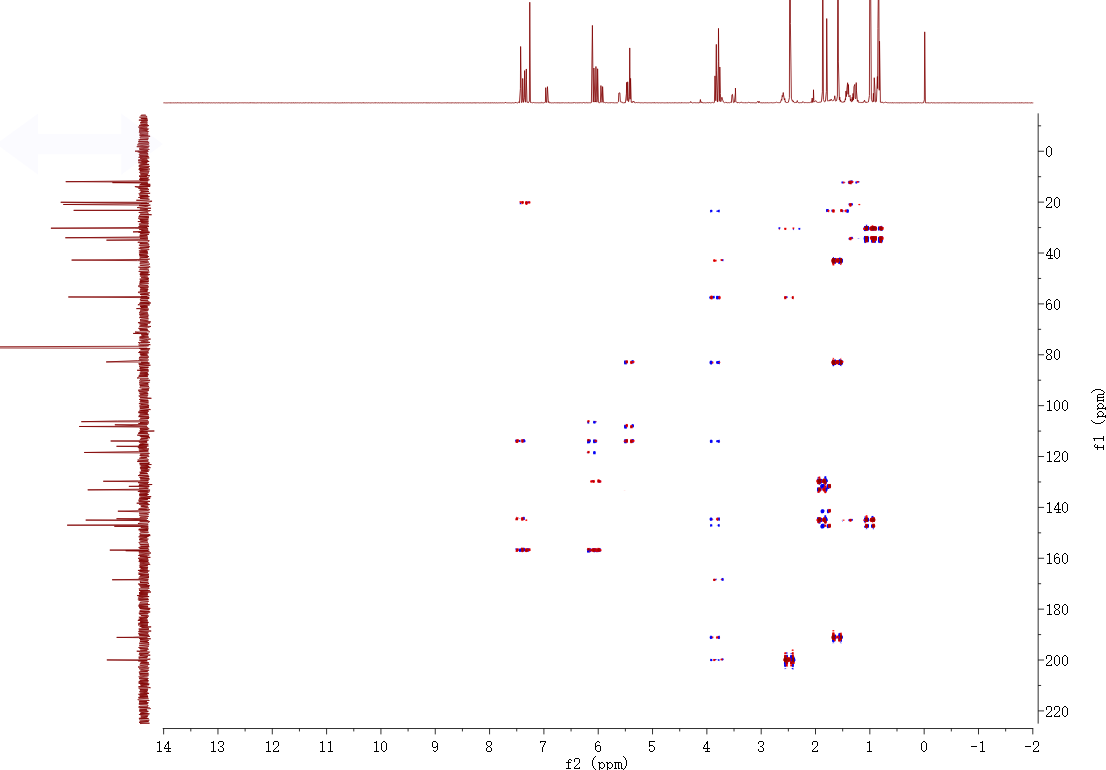
**

**Figure S26.** HMBC (chloroform-*d*) spectrum of compound **4**

**
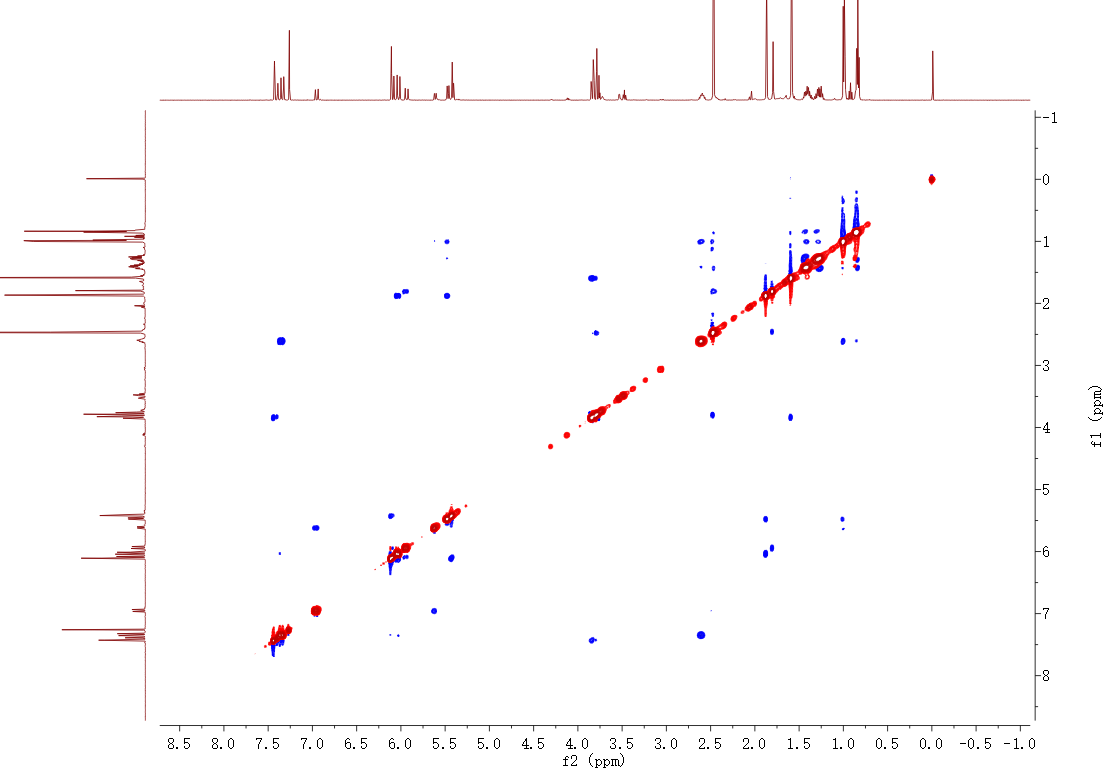
**

**Figure S27.** NOESY (chloroform-*d*) spectrum of compound **4**

**Figure S28.** HRESIMS spectrum of compound **4**

**
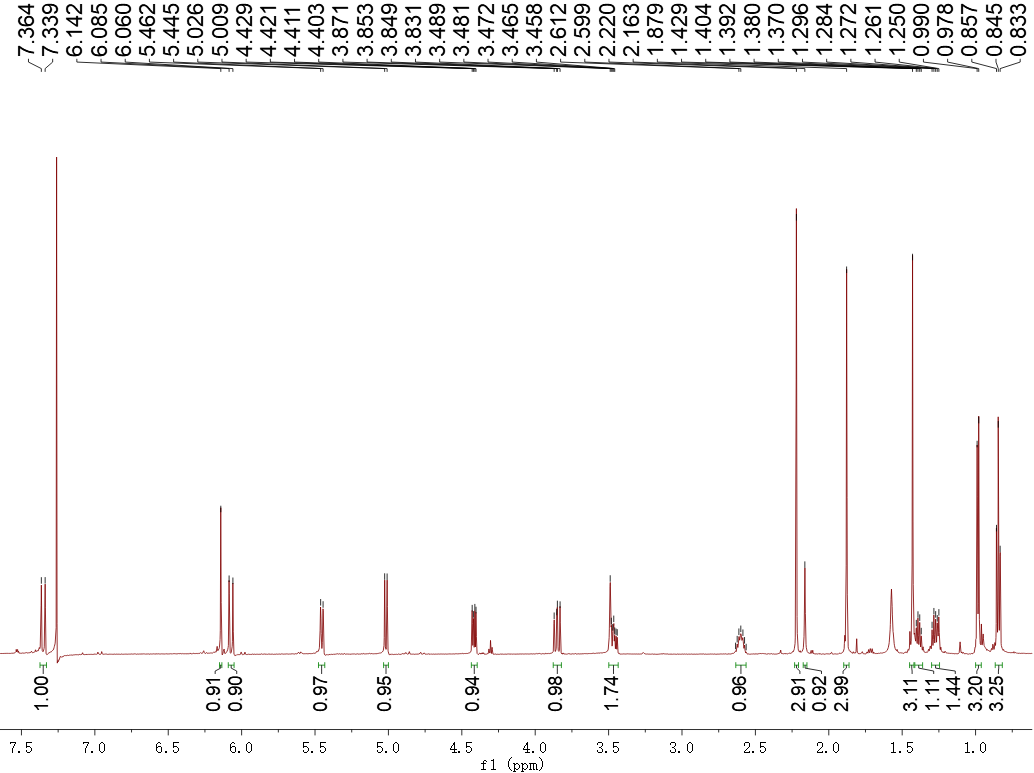
**

**Figure S29.** ^1^H NMR (600 MHz, chloroform-*d*) spectrum of compound **5**

**
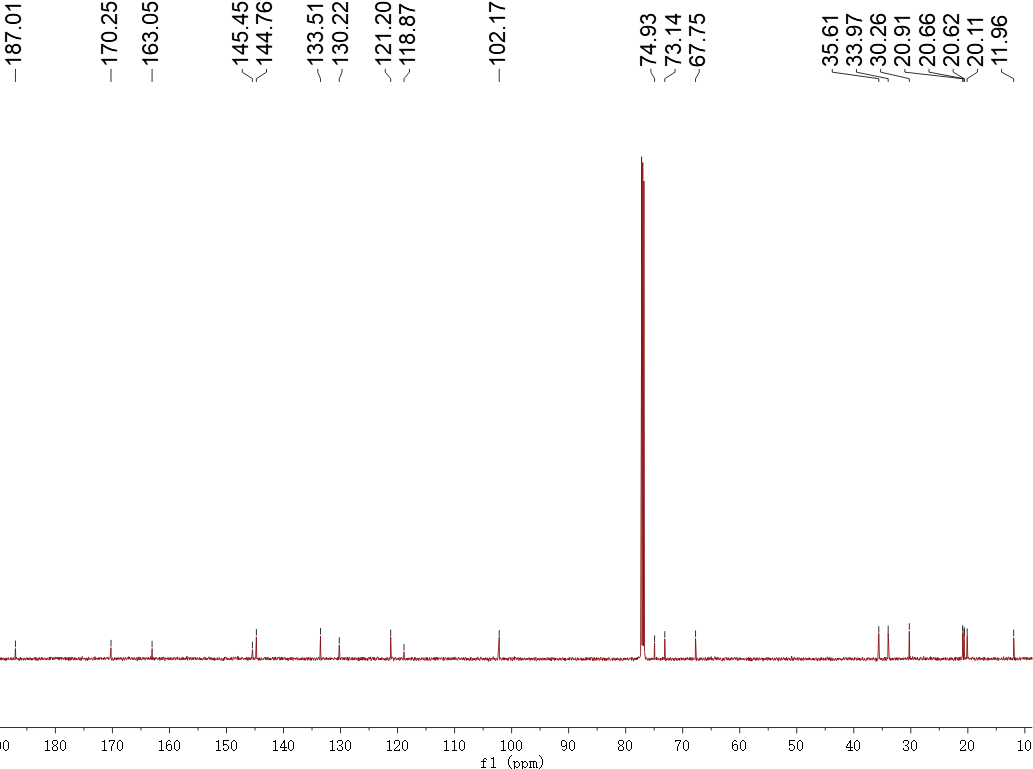
**

**Figure S30.** ^13^C NMR (150 MHz, chloroform-*d*) spectrum of compound **5**

**
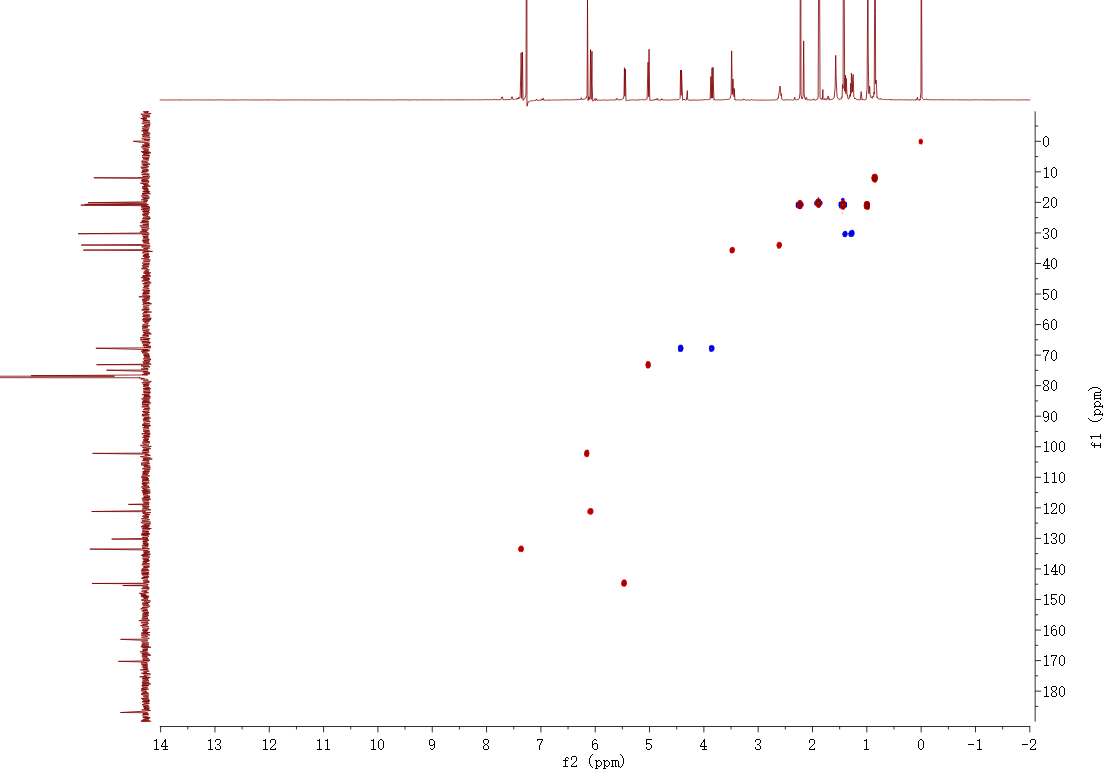
**

**Figure S31.** HSQC (chloroform-*d*) spectrum of compound **5**

**
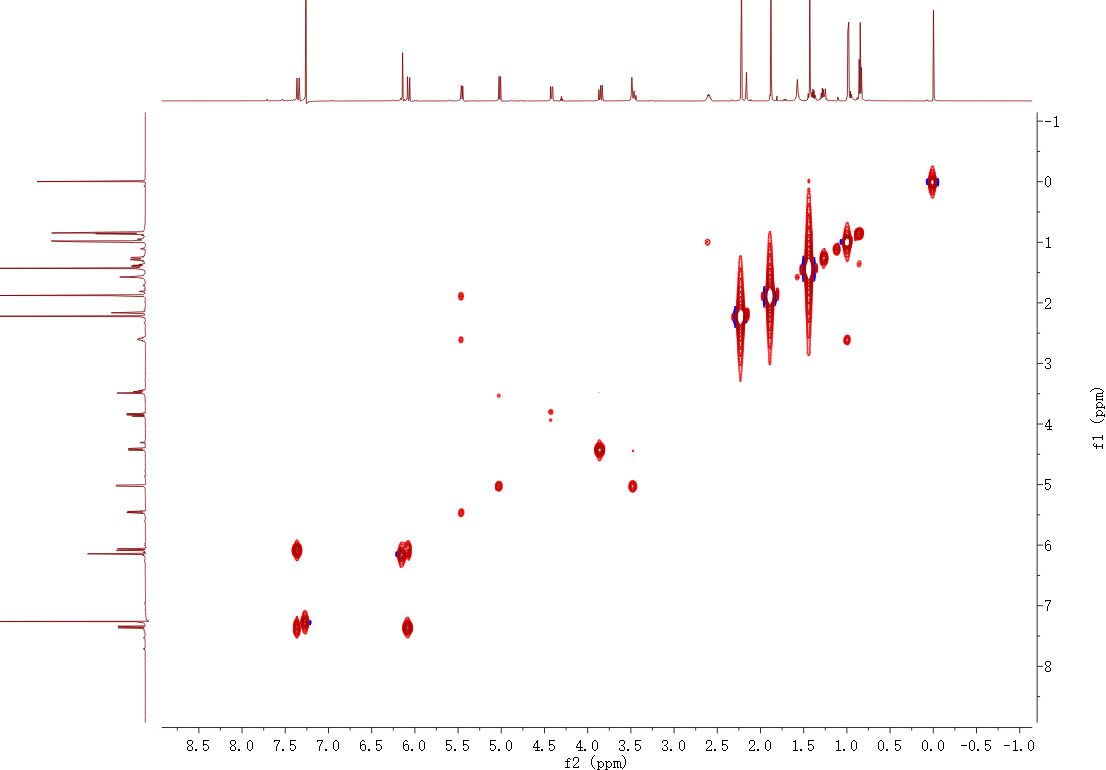
**

**Figure S32.** ^1^H-^1^H COSY (chloroform-*d*) spectrum of compound **5**

**
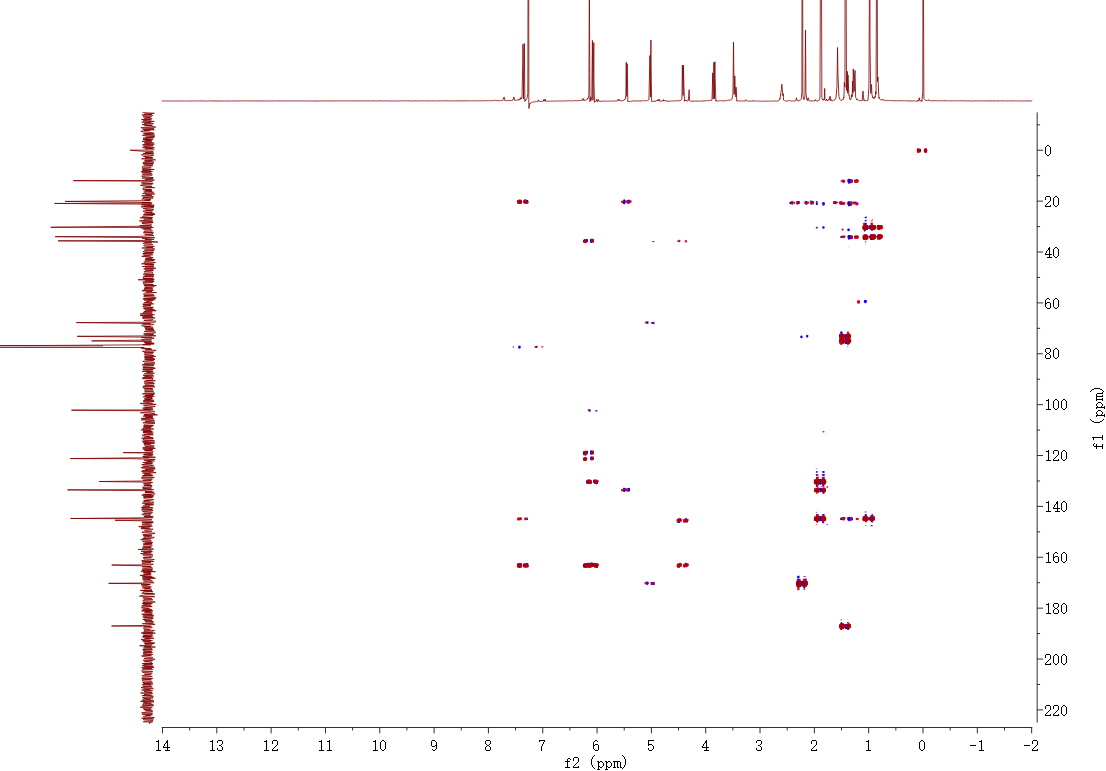
**

**Figure S33.** HMBC (chloroform-*d*) spectrum of compound **5**

**
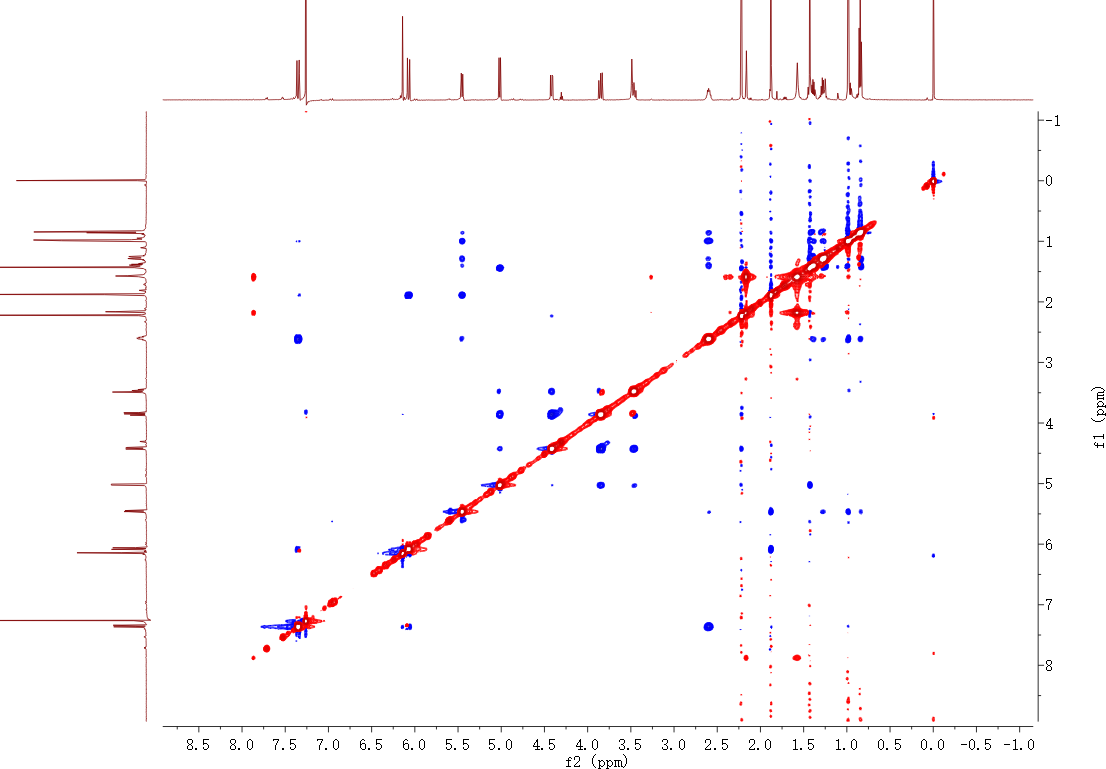
**

**Figure S34.** NOESY (chloroform-*d*) spectrum of compound **5**

**Figure S35.** HRESIMS spectrum of compound **5**

**
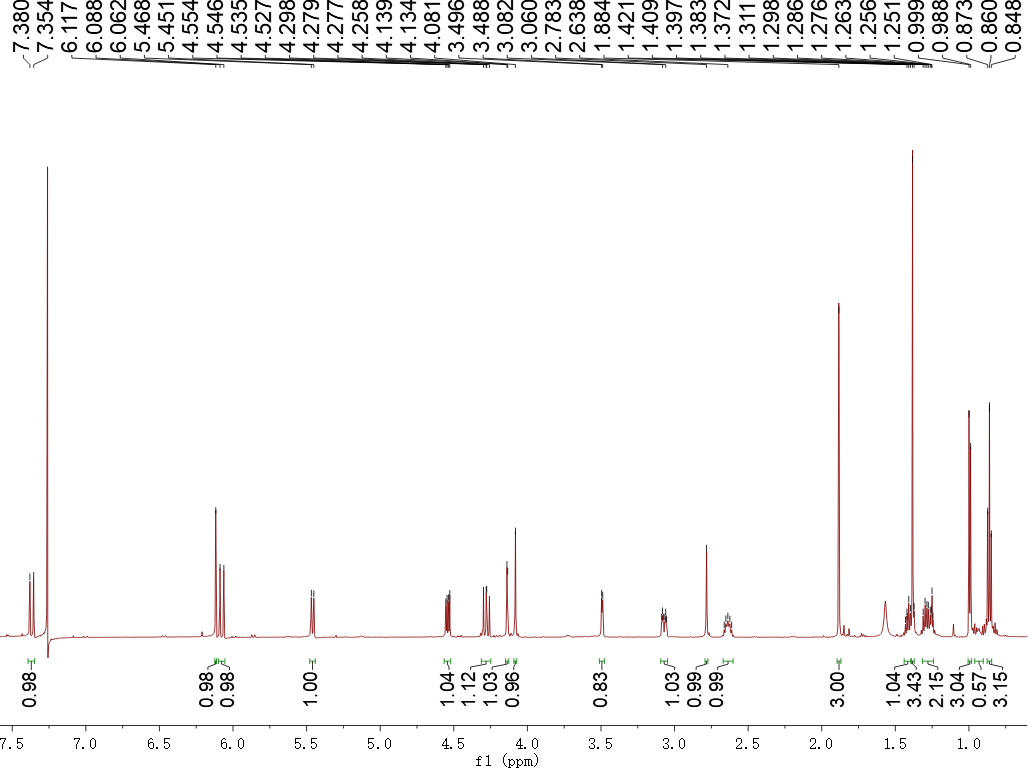
**

**Figure S36.** ^1^H NMR (600 MHz, chloroform-*d*) spectrum of compound **6**

**
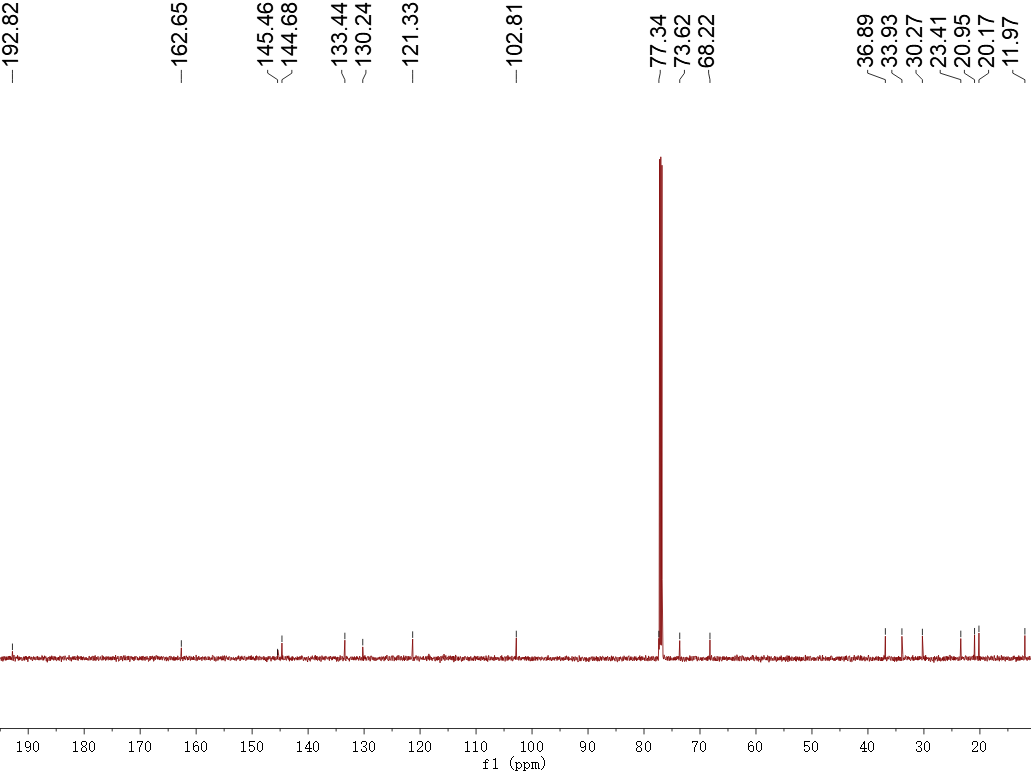
**

**Figure S37.** ^13^C NMR (150 MHz, chloroform-*d*) spectrum of compound **6**

**
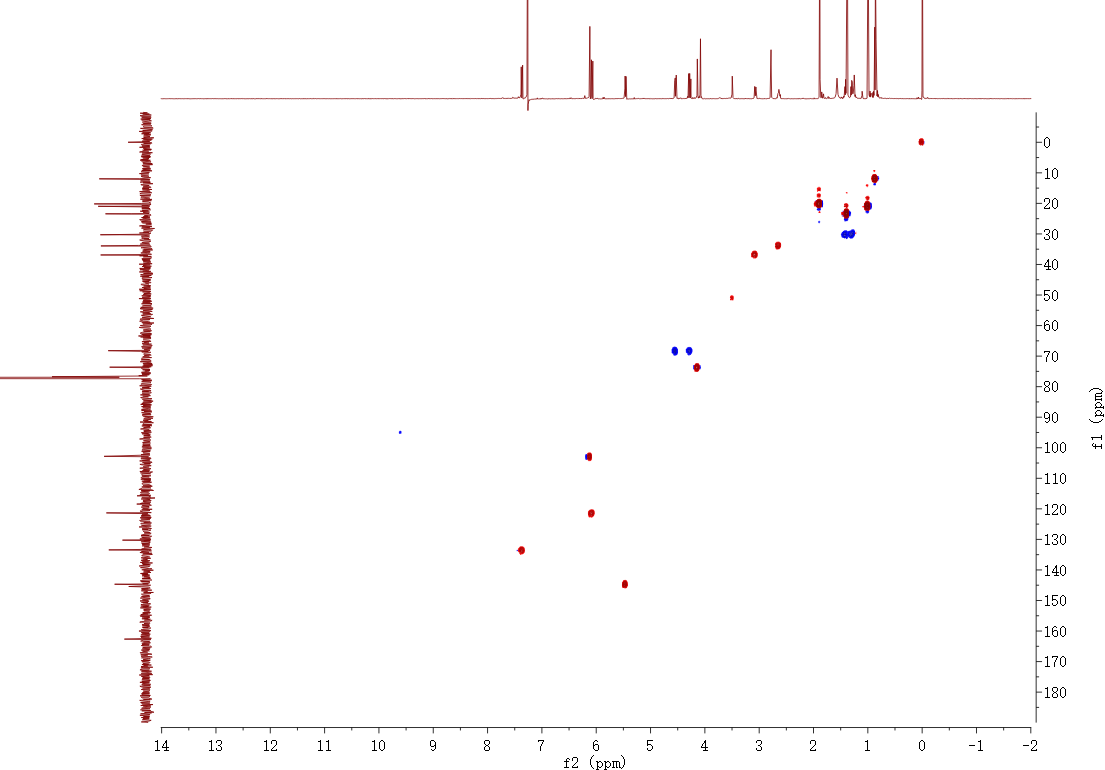
**

**Figure S38.** HSQC (chloroform-*d*) spectrum of compound **6**

**
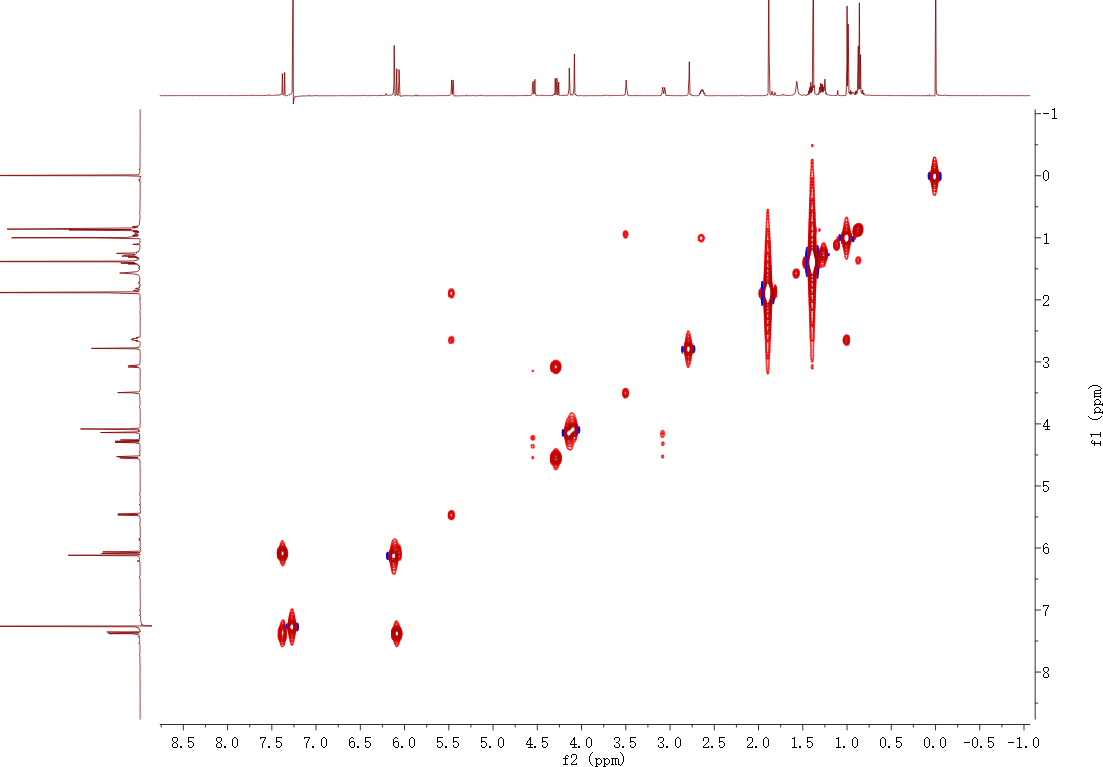
**

**Figure S39.** ^1^H-^1^H COSY (chloroform-*d*) spectrum of compound **6**

**
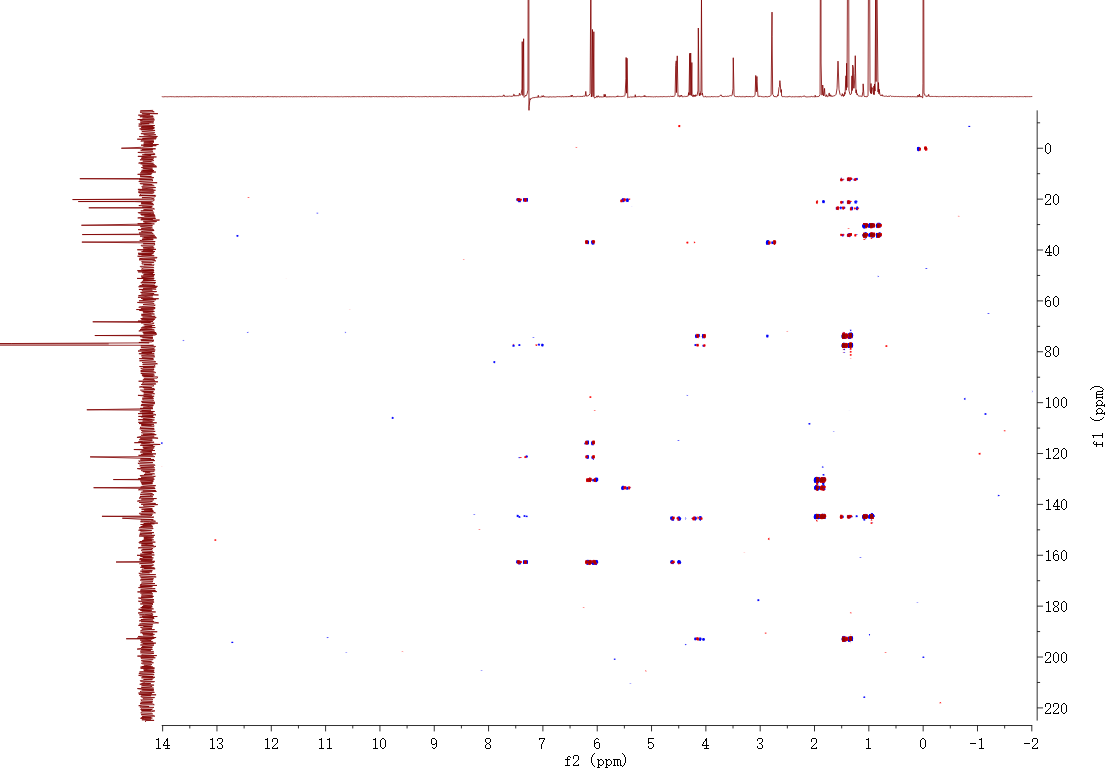
**

**Figure S40.** HMBC (chloroform-*d*) spectrum of compound **6**

**
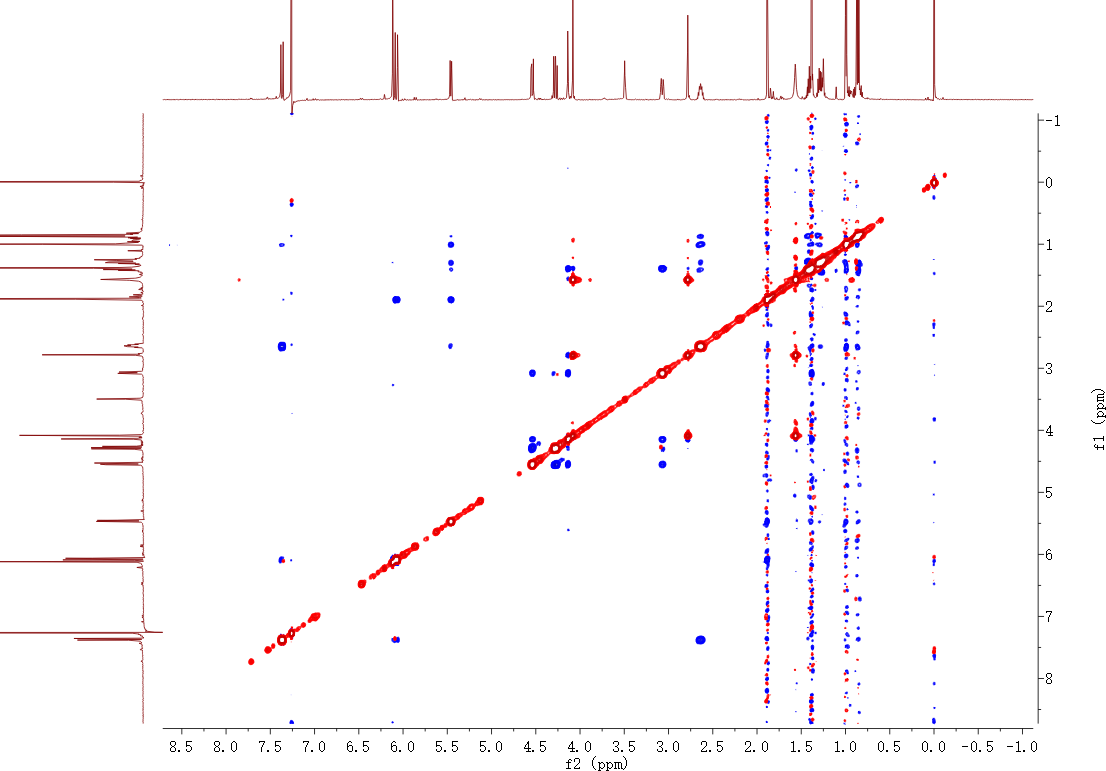
**

**Figure S41.** NOESY (chloroform-*d*) spectrum of compound **6**

**Figure S42.** HRESIMS spectrum of compound **6**

**Figure S43** Regression equation of inhibition rate of compound **3** against the growth of *A. retroflexus* L.

**Figure S44** Regression equation of inhibition rate of compound **4** against the growth of *A. retroflexus* L.

**Figure S45** Regression equation of inhibition rate of compound **7** against the growth of *A. retroflexus* L.

**Figure S46** Regression equation of inhibition rate of glufosinate ammonium against the growth of *A. retroflexus* L.

**Table S47** The germination effect of *A. retroflexus* L. seeds inhibited by different concentrations of compound **3**

| conc. (μg mL⁻^1^) | plumule length (cm) | plumule inhibition rate (%) | radicle length (cm) | radicle inhibition rate (%) |
| --- | --- | --- | --- | --- |
| 500 | 0.08 ± 0.02 | 91.83 | 0.10 ± 0.02 | 96.08 |
| 250 | 0.16 ± 0.02 | 84.01 | 0.21 ± 0.01 | 91.55 |
| 125 | 0.49 ± 0.03 | 50.79 | 0.97 ± 0.02 | 61.32 |
| 62.5 | 0.81 ± 0.01 | 19.42 | 1.74 ± 0.05 | 30.51 |
| 31.25 | 0.95 ± 0.03 | 5.20 | 2.14 ± 0.04 | 14.37 |
| CK | 1.00 ± 0.06 | − | 2.50 ± 0.05 | − |

**Table S48** The germination effect of *A. retroflexus* L. seeds inhibited by different concentrations of compound **4**

| conc. (μg mL⁻^1^) | plumule length (cm) | plumule inhibition rate (%) | radicle length (cm) | radicle inhibition rate (%) |
| --- | --- | --- | --- | --- |
| 500 | 0.07 ± 0.03 | 92.52 | 0.07 ± 0.02 | 97.11 |
| 250 | 0.13 ± 0.01 | 87.25 | 0.21 ± 0.01 | 91.43 |
| 125 | 0.40 ± 0.01 | 60.14 | 0.86 ± 0.02 | 65.54 |
| 62.5 | 0.66 ± 0.04 | 33.61 | 1.48 ± 0.02 | 40.83 |
| 31.25 | 0.84 ± 0.01 | 15.58 | 1.87 ± 0.02 | 25.01 |
| CK | 1.00 ± 0.06 | − | 2.50 ± 0.05 | − |

**Table S49** The germination effect of *A. retroflexus* L. seeds inhibited by different concentrations of compound **7**

| conc. (μg mL⁻^1^) | plumule length (cm) | plumule inhibition rate (%) | radicle length (cm) | radicle inhibition rate (%) |
| --- | --- | --- | --- | --- |
| 500 | 0.12 ± 0.01 | 88.02 | 0.17 ± 0.03 | 93.21 |
| 250 | 0.17 ± 0.02 | 82.64 | 0.31 ± 0.03 | 87.43 |
| 125 | 0.45 ± 0.05 | 55.02 | 1.01 ± 0.01 | 59.54 |
| 62.5 | 0.73 ± 0.04 | 26.83 | 1.65 ± 0.01 | 33.83 |
| 31.25 | 0.89 ± 0.04 | 10.92 | 1.90 ± 0.02 | 23.92 |
| CK | 1.00 ± 0.06 | − | 2.50 ± 0.05 | − |

**Table S50** The germination effect of *A. retroflexus* L. seeds inhibited by different concentrations of glufosinate ammonium

| conc. (μg mL⁻^1^) | plumule length (cm) | plumule inhibition rate (%) | radicle length (cm) | radicle inhibition rate (%) |
| --- | --- | --- | --- | --- |
| 500 | 0.19 ± 0.02 | 81.21 | 0.35 ± 0.03 | 86.01 |
| 250 | 0.24 ± 0.01 | 76.32 | 0.45 ± 0.03 | 81.92 |
| 125 | 0.48 ± 0.02 | 52.08 | 1.12 ± 0.01 | 55.08 |
| 62.5 | 0.72 ± 0.02 | 28.01 | 1.65 ± 0.03 | 34.01 |
| 31.25 | 0.89 ± 0.02 | 11.34 | 2.08 ± 0.05 | 17.00 |
| CK | 1.00 ± 0.06 | − | 2.50 ± 0.05 | − |

**Figure S51** Regression equation of inhibition rate of compound **4** against the growth of *Abutilon theophrasti* Medikus

**Figure S52** Regression equation of inhibition rate of compound **7** against the growth of *Abutilon theophrasti* Medikus

**Figure S53** Regression equation of inhibition rate of glufosinate ammonium against the growth of *Abutilon theophrasti* Medikus

**Table S54** The germination effect of *Abutilon theophrasti* Medikus seeds inhibited by different concentrations of compound **4**

| conc. (μg mL⁻^1^) | plumule length (cm) | plumule inhibition rate (%) | radicle length (cm) | radicle inhibition rate (%) |
| --- | --- | --- | --- | --- |
| 500 | 0.77 ± 0.07 | 51.27 | 0.35 ± 0.03 | 51.09 |
| 250 | 0.91 ± 0.04 | 42.63 | 0.44 ± 0.02 | 39.55 |
| 125 | 1.09 ± 0.03 | 30.77 | 0.53 ± 0.02 | 26.71 |
| 62.5 | 1.23 ± 0.02 | 22.08 | 0.58 ± 0.01 | 19.08 |
| 31.25 | 1.36 ± 0.03 | 13.63 | 0.64 ± 0.02 | 11.63 |
| CK | 1.58 ± 0.04 | − | 0.72 ± 0.05 | − |

**Table S55** The germination effect of *Abutilon theophrasti* Medikus seeds inhibited by different concentrations of compound **7**

| conc. (μg mL⁻^1^) | plumule length (cm) | plumule inhibition rate (%) | radicle length (cm) | radicle inhibition rate (%) |
| --- | --- | --- | --- | --- |
| 500 | 0.51 ± 0.02 | 67.72 | 0.36 ± 0.01 | 50.06 |
| 250 | 0.93 ± 0.03 | 41.38 | 0.46 ± 0.03 | 35.83 |
| 125 | 1.14 ± 0.01 | 27.95 | 0.57 ± 0.04 | 20.95 |
| 62.5 | 1.27 ± 0.02 | 19.78 | 0.64 ± 0.02 | 11.78 |
| 31.25 | 1.39 ± 0.05 | 12.27 | 0.67 ± 0.03 | 6.27 |
| CK | 1.58 ± 0.04 | − | 0.72 ± 0.05 | − |

**Table S56** The germination effect of *Abutilon theophrasti* Medikus seeds inhibited by different concentrations of glufosinate ammonium

| conc. (μg mL⁻^1^) | plumule length (cm) | plumule inhibition rate (%) | radicle length (cm) | radicle inhibition rate (%) |
| --- | --- | --- | --- | --- |
| 500 | 0.18 ± 0.05 | 88.61 | 0.13 ± 0.02 | 82.28 |
| 250 | 0.53 ± 0.02 | 66.46 | 0.28 ± 0.04 | 61.11 |
| 125 | 0.77 ± 0.02 | 51.56 | 0.40 ± 0.01 | 44.44 |
| 62.5 | 0.88 ± 0.04 | 44.03 | 0.47 ± 0.06 | 35.09 |
| 31.25 | 0.98 ± 0.06 | 38.21 | 0.53 ± 0.02 | 26.45 |
| CK | 1.58 ± 0.04 | − | 0.72 ± 0.05 | − |
